# The RIPK3-IL-6 axis mediates kidney injury in cytokine storm syndrome

**DOI:** 10.1038/s41419-026-08686-1

**Published:** 2026-04-15

**Authors:** Juan Guerrero-Mauvecin, Diego Martin-Sánchez, Natalia Villar-Gómez, Susana Carrasco, Blanca Chinchilla, Sol Carriazo, Julio M. Martinez-Moreno, Lucia Miño-Izquierdo, Manuel J. Gómez, Maria D. Sanchez-Niño, Alberto Ortiz, Ana B. Sanz

**Affiliations:** 1 Laboratorio de Nefrología Experimental, Instituto de Investigación Sanitaria-Fundacion Jimenez Diaz (IIS-FJD), Universidad Autonoma de Madrid, Madrid, Spain; 2 Servicio Oncología Médica, Hospital Universitario Virgen del Rocío, Sevilla, Spain; 3 RICORS2040, Madrid, Spain; 4 VISAVET Health Surveillance Centre, Complutense University of Madrid, Madrid, Spain; 5 Department of Animal Production, Faculty of Veterinary Medicine, Complutense University of Madrid, Madrid, Spain; 6 Division of Nephrology, University Health Network, Toronto, ON Canada; 7 Cobiomic Bioscience SL. EBT University of Cordoba/IMIBIC, Cordoba, Spain; 8 Unidad de Bioinformatica, Centro Nacional de Investigaciones Cardiovasculares (CNIC), Madrid, Spain; 9 Department of Pharmacology, Universidad Autonoma de Madrid, Madrid, Spain; 10 IRSIN, Madrid, Spain; 11 Department of Medicine, Universidad Autonoma de Madrid, Madrid, Spain

**Keywords:** Mechanisms of disease, Acute kidney injury

## Abstract

Receptor-interacting protein kinase 3 (RIPK3) is best known as a mediator of necroptosis, a regulated necrosis pathway observed in diseases with high inflammatory components. We explored the role of RIPK3 in cytokine storm syndrome (CSS)-induced acute kidney injury induced by lipopolysaccharide (LPS) in wild-type and *Ripk3-*deficient mice. For the in vitro experiments, we treated both primary tubular epithelial cells and bone marrow-derived cells from wild-type and *Ripk3*-deficient mice with LPS. *Ripk3* deficiency improved renal function and increased survival in CSS, a common condition observed in severe infections and other scenarios, and protection was associated with reduced inflammation. Mechanistically, the necroptosis pathway did not play a key role in kidney protection by Ripk3 deficiency, and the NLRP3 inflammasome was partially implicated. Olink plasma proteomics identified IL-6 as the most upregulated inflammatory protein in the kidney and circulation, and it was most responsive to *Ripk3* deficiency. *Ripk3*-deficient mice had suppressed kidney, liver, and lung *Il-6* expression as well as suppressed kidney activation of STAT3, a transcription factor downstream of IL-6. In bone marrow chimeric mice, RIPK3-expressing bone marrow-derived cells were required to drive IL-6 expression, and AKI and kidney *Il-6* expression correlated with loss of renal function. In this regard, in vitro experiments have shown that RIPK3 mediates *Il-6* expression in bone marrow cells but not in tubular cells. Additionally, targeting the IL-6 receptor improves kidney function and reduces kidney inflammation in CSS-AKI. Kidney transcriptomic data of human AKI associated with COVID-19 CSS were consistent with activation of the RIPK3-IL-6 axis. The RIPK3-IL-6 axis in bone marrow cells mediates systemic inflammation and kidney injury induced by a cytokine storm, independent of the necroptosis and inflammasome pathways. Specific targeting of bone marrow RIPK3 may limit kidney inflammation, without the potential adverse effects of systemic RIPK3 targeting.

## Introduction

Acute kidney injury (AKI) is characterized by a sudden and usually transient loss of kidney function [1]. The incidence of AKI is increasing as the aging population undergoes complex medical procedures. Patients with AKI have a higher risk of developing chronic kidney disease (CKD) and kidney failure [2]. Mortality rates reach up to 50%, and even a short-timed injury contributes to persistently higher mortality [3]. AKI is a highly heterogeneous condition in terms of etiology, pathophysiology, and kidney outcomes. Currently, no satisfactory treatment attenuates AKI or accelerates recovery [3].

Cytokine storm syndrome (CSS) is defined as a massive immune response characterized by the release of inflammatory mediators, such as cytokines and chemokines, which can lead to multiorgan dysfunction, including AKI, and even death [4]. The main cause of CSS is sepsis, but it can also be triggered by viral infection, endotoxins, and sterile inflammatory processes such as CAR-T cell therapy [4, 5]. Sepsis is also the primary cause of AKI in critically ill patients, and it is commonly associated with multiorgan dysfunction, high morbidity, and mortality [6]. CSS during SARS-CoV-2 infection may contribute to the high incidence of AKI in patients with COVID-19 [7–9]. More recently, CAR-T therapy has also been associated with a high incidence of AKI, and CAR-T cell-induced CSS appears to be a contributing factor [10]. Therefore, CSS may promote AKI in several clinical scenarios. Interleukin-6 (IL-6) is a pivotal cytokine released during CSS, and different treatments aimed at IL-6 signaling have been approved for autoimmune disorders and CSS [11]. IL-6 may also mediate kidney injury associated with CSS, since serum IL-6 increases in sepsis-induced AKI and contributes to severe COVID-19 [4, 6, 7].

Receptor-interacting protein 3 (RIPK3) plays a key role in necroptosis, a pathway of regulated necrosis that contributes to kidney injury, in several preclinical models of AKI [12]. However, RIPK3 may induce a cell-death-independent inflammatory response in murine colitis, where it mediates NF-κB activation and inflammatory cytokine expression [13]. Similar results have been observed in murine abdominal aortic aneurysm [14]. RIPK3 has also been associated with inflammasome activation [15]. Regarding AKI, urinary RIPK3 levels are higher in septic patients than in non-septic patients, and septic patients with AKI have higher urinary and plasma RIPK3 levels than those without AKI [16]. Additionally, RIPK3-deficient mice preserved kidney function during cecal ligation and puncture-induced severe infection and sepsis. However, the inflammatory response was not evaluated, and the relative contribution of improved antibacterial defenses versus interference with inflammation was not addressed [16].

We previously demonstrated that in nephrotoxic folic acid-AKI, RIPK3 promotes inflammation independent of cell death, and this is mediated by bone marrow (BM)-derived cells [17]. We now report that in LPS-induced endotoxemia (i.e., sterile CSS), RIPK3 from BM cells contributes to systemic inflammation and the resulting AKI through the recruitment of the RIPK3-IL-6 axis. In this regard, IL-6 targeting reproduced the molecular and kidney-protective features of RIPK3 deficiency. These findings identified the RIPK3-IL-6 axis as a therapeutic target for CSS and AKI.

## Results

### RIPK3 deficiency improves renal function and mice survival in CSS-AKI

**Fig. 1:**
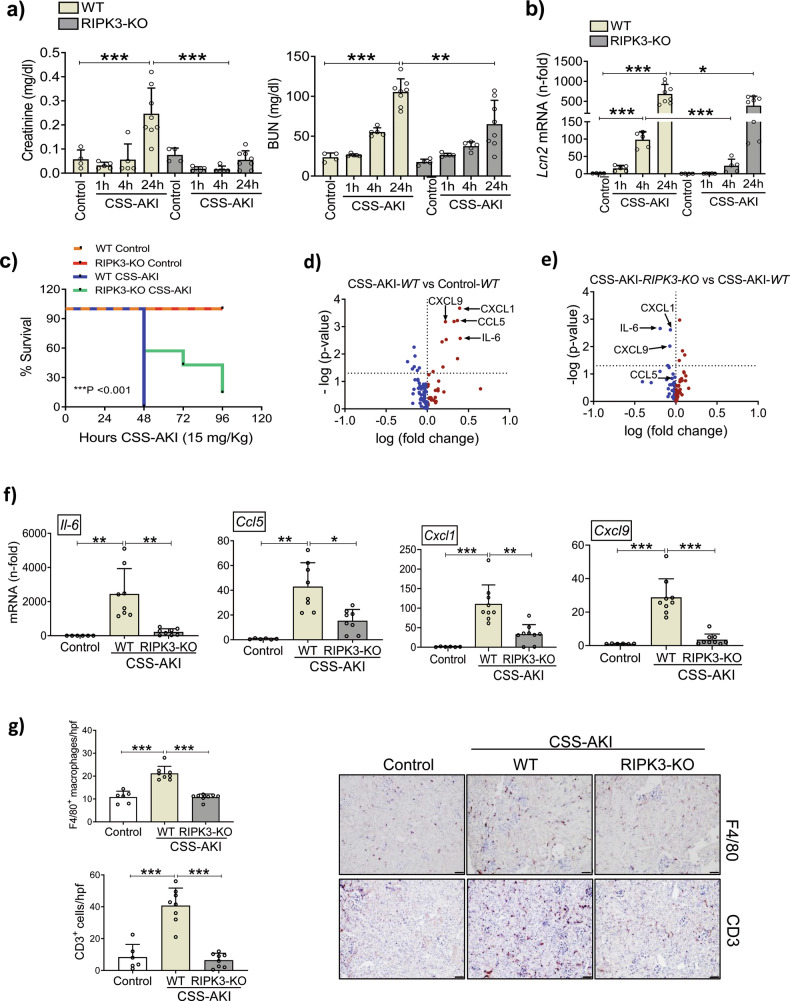
*Ripk3* deficiency preserved renal function, increased survival, and has a local anti-inflammatory effect after CSS-AKI. CSS-AKI was induced by a single LPS injection, and mice were sacrificed at 1, 4, and 24 h. **a** Renal function assessed by plasma creatinine and BUN levels in WT and *Ripk3*-KO mice with CSS-AKI. **b** Kidney expression of *Lcn2*, which encodes NGAL, is reduced in *Ripk3*-KO mice compared with WT mice with CSS-AKI. **c** Survival of *Ripk3*-deficient and WT mice after CSS-AKI induction (*n* = 7 animals per group). **d**, **e** Volcano plots of the Olink protein panel in kidney tissue 24 h after LPS injection. **d** CSS-AKI/Control ratio. IL-6 is the most increased protein in CSS-AKI at 24 h compared with control mice. **e** CSS-AKI: *Ripk3*-KO/WT ratio. IL-6 is the most decreased protein in *Ripk3*-KO mice compared with WT mice. **f** Kidney *Il-6, Ccl5, Cxcl1*, and *Cxcl9* mRNA levels are lower in *Ripk3*-KO than in WT mice in CSS-AKI at 24 h. **g** Kidney interstitial infiltration by F4/80 positive interstitial macrophages and CD3+ T cells in CSS-AKI 24 h after injury. Representative images (Magnification 200×) and quantification. Scale 50 µm. **a**, **b**, **f**, **g** Mean ± SD of 6–8 animals per group. *p < 0.05, **p < 0.01; ***p < 0.001.

First, we addressed the impact of RIPK3 on kidney function and injury in mice with CSS-AKI. CSS-AKI is characterized by mild histological injury but high mortality (Figs. S1 and 1c). *Ripk3*-KO mice had better preserved renal function, as indicated by lower serum creatinine and BUN levels 24 h after injury induction (Fig. 1a). Moreover, *Ripk3*-KO mice showed reduced expression of *Lcn2*, the gene encoding neutrophil gelatinase-associated lipocalin (NGAL), a well-established marker of tubular injury and AKI (Fig. 1b). These mice also exhibited improved survival compared to WT mice (Fig. 1c). These results suggest that RIPK3 contributes to kidney dysfunction and mortality in CSS-AKI patients.

### *Ripk3* deficiency reduces kidney cytokine storm and interstitial inflammation in CSS-AKI

**Fig. 2:**
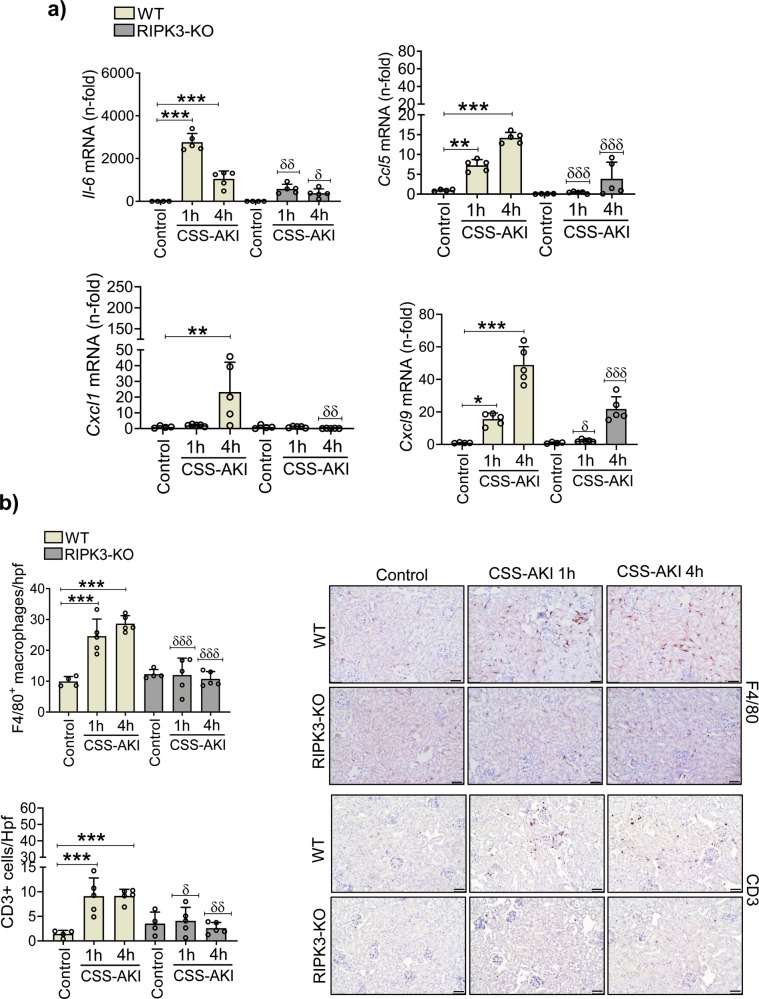
*Ripk3* deficiency prevents the early kidney inflammatory response in CSS-AKI. CSS-AKI was induced by LPS injection, and mice were sacrificed at 1 and 4 h**. a** Kidney *Il-6, Ccl5, Cxcl1*, and *Cxcl9*, mRNA levels, measured by RT-PCR, are lower in *Ripk3*-KO than in WT mice in CSS-AKI at 1 and 4 h. **b** Kidney interstitial infiltration by F4/80 positive interstitial macrophages and CD3+ T cells in CSS-AKI at 1 and 4 h. The inflammatory infiltrate is reduced in *Ripk3*-KO mice. Representative images (Magnification 200x) and quantification. Scale 50 µm. **a**, **b** Mean ± SD of 4–5 animals per group. *p < 0.05; **p < 0.01; ***p < 0.001; ^ẟ^*p*<0.05 vs CSS-AKI WT; ^ẟẟ^*p* < 0.01 vs CSS-AKI WT; ^ẟẟẟ^*p* < 0.001 vs CSS-AKI WT.

As RIPK3 contributes to kidney injury and failure in CSS-AKI, we addressed its impact on kidney inflammation. Using a non-biased approach, kidney Olink proteomics identified IL-6 as the most upregulated cytokine in CSS-AKI WT kidneys at 24 h (Table 1, Fig. 1d). Additionally, Olink proteomics revealed that CXCL1, CXCL9, and CCL5 were increased in CSS-AKI (Fig. 1d). Gene expression analysis assessed by RT-PCR confirmed a large upregulation of kidney *Il-6* expression in CSS-AKI, several-fold higher than those other proinflammatory cytokines identified as upregulated by Olink (e.g., *Ccl5, Cxcl1*, and *Cxl9*) (Fig. 1f). Increased expression of kidney inflammatory genes was associated with kidney leukocyte infiltration (F4/80+ macrophages, CD3+ lymphocytes) (Fig. 1g). A significant upregulation of kidney *Il-6* expression was noted as early as 1 h following LPS administration, in association with a milder and slower elevation of other cytokines and chemokines (Fig. 2a) and early leukocyte infiltration (Fig. 2b).

**Table 1:** Olink kidney proteomics.

Protein	CSS-AKI WT vs control (fold-change)	*p* value	CSS-AKI Ripk3-KO vs CSS-AKI WT (fold-change)	*p* value
IL-6	2.51	0.000	0.64	0.002
CXCL1	2.47	0.000	0.86	0.002
TNFRSF12A	2.33	0.027	0.64	0.080
CCL5	2.32	0.000	0.90	0.145
CCL3	2.11	0.000	0.99	0.789
CCL20	1.69	0.001	0.96	0.497
CXCL9	1.66	0.000	0.84	0.010
CSF2	1.55	0.015	1.13	0.364
CCL2	1.51	0.001	1.04	0.466
CA13	1.18	0.034	0.92	0.101
LGMN	1.04	0.022	0.98	0.276

Proteins upregulated at 24 h with a *p* value < 0.05 in CSS-AKI WT vs control are shown; *p* values < 0.05 are marked in red for CSS-AKI WT vs control and in blue for CSS-AKI Ripk3-KO vs CSS-AKI WT.

*Ripk3*-KO mice are protected from CSS-AKI-associated kidney inflammation. Olink proteomics identified IL-6 as the most downregulated and differentially expressed cytokine at 24 h in CSS-AKI *Ripk3*-KO mice compared with CSS-AKI WT kidneys (Table 1, Fig. 1e). Additionally, CCL5, CXCL1, and CXCL9 were downregulated in CSS-AKI *Ripk3*-KO mice compared to CSS-AKI WT mice (Table 1, Fig. 1e). Downregulation of kidney *Il-6, Ccl5, Cxcl1*, and *Cxcl9* in *Ripk3*-KO mice with CSS-AKI compared to WT mice was already noted at 1 h and persisted up to 24 h (Figs. 1f and 2a) and was associated with milder kidney inflammatory cell infiltrates (Figs. 1g and 2b).

### Neither necroptosis nor inflammasome are involved in CSS-AKI

RIPK3 is a key component of the necroptosis pathway along with RIPK1 and MLKL. To address whether necroptosis is involved in CSS-AKI, we pretreated mice with Nec-1, a RIPK1 inhibitor. Nec-1 did not alter kidney function and had a mild anti-inflammatory effect (Fig. S2a, b). However, kidney *Il6* expression in CSS-AKI mice pretreated with Nec-1 was still over 1000-fold higher than in control mice. These findings do not support a key role of necroptosis in CSS-AKI.

NLRP3 inflammasome has also been associated with RIPK3-induced inflammation. In this regard, kidney NLRP3 and cleaved caspase-1 (caspase-1 p20) levels increased in CSS-AKI at early time points, and the increase in kidney NLRP3 was sensitive to *Ripk3* deficiency (Fig. S3a, b). Additionally, NLRP3 deficiency prevented caspase-1 activation, but did not prevent kidney failure (Fig. S3c, d). Regarding the cytokine storm, NLRP3 deficiency did not prevent *Il6* and *Ccl5* overexpression, but reduced *Cxcl1* and *Cxcl9* expression (Fig. S3e). These results suggest that RIPK3 recruits different pathways to contribute to the cytokine storm phenotype, some of which are dispensable for the protective impact of *Ripk3* deficiency.

### RIPK3 protein is weakly upregulated in kidneys with CSS-AKI

**Fig. 3:**
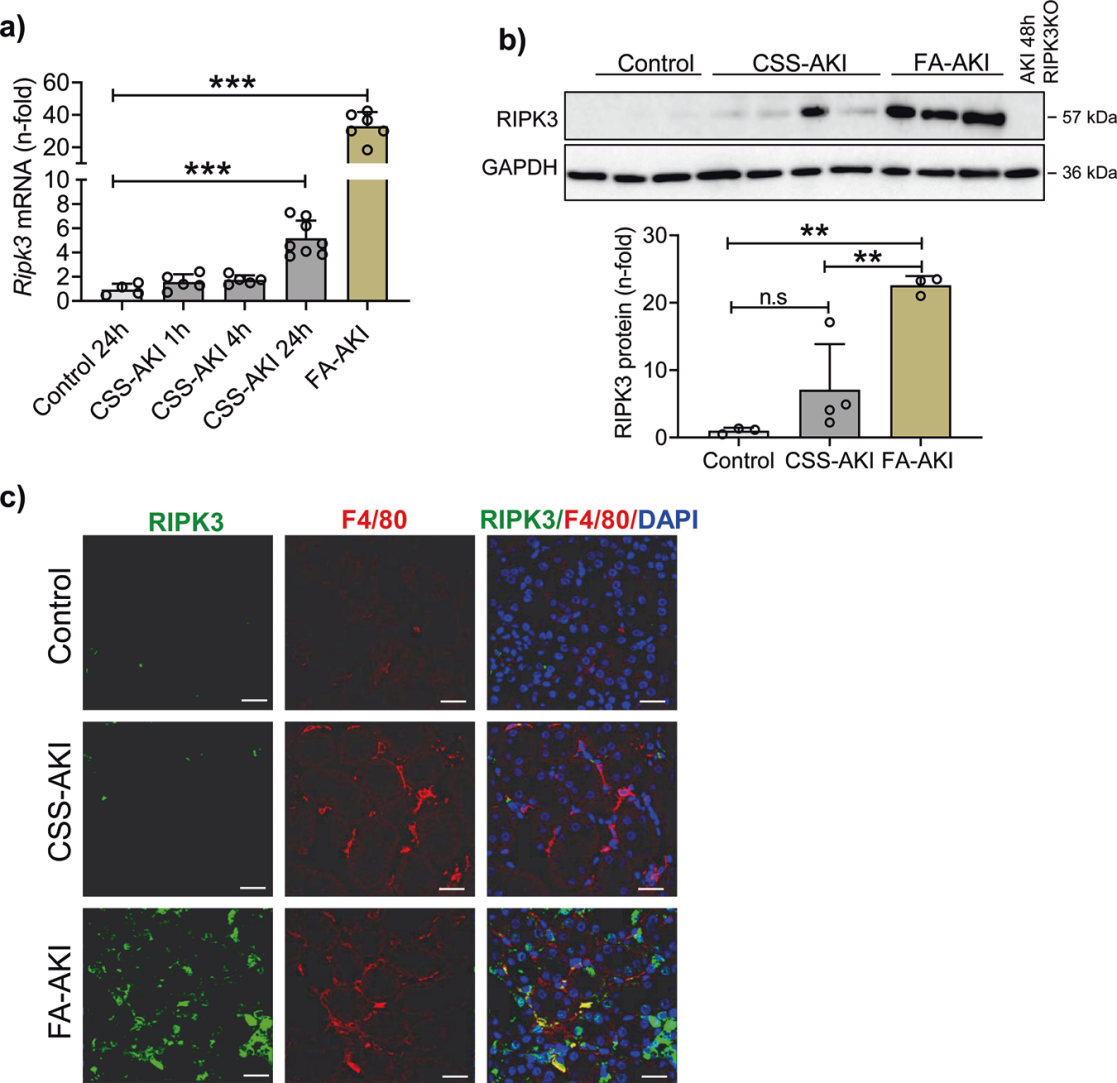
RIPK3 expression in kidneys with CSS-AKI. **a** *Ripk3* mRNA expression was measured by RT-PCR in kidneys with CSS-AKI at 1, 4, and 24 h. Kidneys from mice with folic acid-AKI (FA-AKI) were analyzed as a positive control of RIPK3 overexpression. Mean ± SD of 4–8 animals per group. ***p < 0.001. **b** RIPK3 protein levels were measured by Western blot in control kidneys and in kidneys with CSS-AKI at 24 h. Kidneys with FA-AKI are used as a positive control of RIPK3 overexpression, and RIPK3-KO mice as a negative control. Mean ± SD of 3–4 animals per group. **p < 0.01. **c** RIPK3 immunofluorescence and colocalization with macrophage marker F4/80 in both CSS-AKI at 24 h and FA-AKI at 48 h. RIPK3 expression was low in kidneys with CSS-AKI, whereas in FA-AKI, it was increased in tubules and interstitial macrophages. Representative images are shown. Confocal microscopy. Magnification, 800×. Scale 20 µm.

Kidney *Ripk3* expression started to increase within 1 h of LPS administration and reached statistical significance at 24 h; however, overall expression levels remained several-fold lower than those in Folic Acid-AKI (FA-AKI), where RIPK3 plays a proinflammatory role [17] (Fig. 3a). A similar observation was made for RIPK3 protein, as assessed by western blot and immunofluorescence (Fig. 3b, c). To test whether RIPK3 is expressed in infiltrating macrophages, we co-stained tissue samples with the macrophage marker F4/80. RIPK3 colocalized with infiltrating macrophages in FA-AKI, but not in LPS-AKI (Fig. 3c). These data suggest that RIPK3 may play a systemic role rather than a local role in CSS-AKI.

### RIPK3 promotes systemic inflammation in CSS induced by LPS injection

**Fig. 4:**
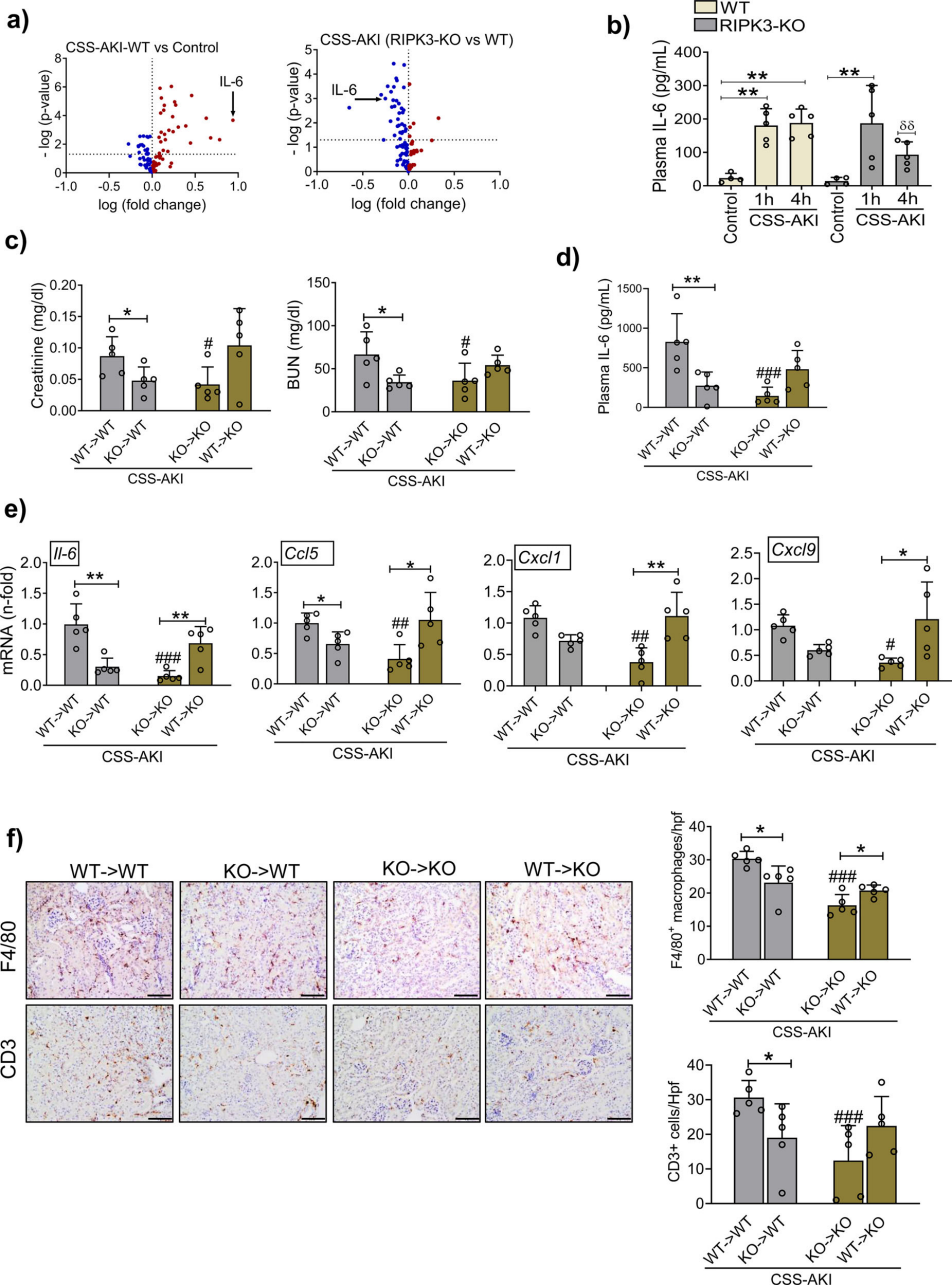
*Ripk3* deficiency reduces systemic inflammation in CSS, and *Ripk3*-KO bone marrow-derived cells prevent renal dysfunction and kidney inflammation during CSS-AKI. **a** Volcano plots of Olink plasma protein panel (3–4 animals per group). CSS-AKI/Control ratio. IL-6 is the most increased protein in the plasma of mice with CSS at 24 h compared with Control mice; CSS-AKI: *Ripk3*-KO/WT ratio. Plasma IL-6 was among the most decreased in *Ripk3*-KO mice compared with WT mice at 24 h after CSS-AKI induction. **b** Plasma levels of IL-6 measured by ELISA at 1 and 4 h. Mean ± SD of 4–5 animals per group. **p < 0.01; ^ẟẟ^*p* < 0.01 vs CSS-AKI WT. **c**–**f** Wild-type (WT) mice with *WT* bone marrow are represented as WT→WT and with *Ripk3*-KO bone marrow as KO→WT. *Ripk3*-KO mice (KO) with *WT* bone marrow are represented as WT→KO and with Ripk3-KO bone marrow as KO→KO. **c** Renal function was assessed by plasma creatinine and BUN levels at 24 h after CSS-AKI induction in bone marrow chimera mice. **d** Plasma levels of IL-6 measured by ELISA at 24 h. **e** Kidney *Il-6, Ccl5, Cxcl1*, and *Cxcl9* mRNA levels assessed by RT-PCR at 24 h of CSS-AKI in bone marrow chimera mice. **f** Kidney interstitial infiltration by F4/80 positive interstitial macrophages and CD3+ T cells in CSS-AKI at 24 h in bone marrow chimera mice. Representative images (Magnification 200×) and quantification. Scale 100 µm. **c**–**f** Mean ± SD of 5 mice per group. *p < 0.05; **p < 0.01; #*p* < 0.05 vs WT->WT; ##*p* < 0.01 vs WT->WT; ###*p* < 0.001 vs WT->WT.

The effect of *Ripk3* deficiency on systemic cytokine storm was also analyzed. Olink proteomics revealed a severe systemic proinflammatory response induced by LPS at 24 h, in which 27/92 (29%) inflammatory proteins were upregulated. Of these, the majority (18/27, 67%) were *Ripk3* deficiency-responsive, i.e., they were significantly less upregulated in *Ripk3*-deficient mice (Table 2, Fig. 4a). As in the kidney, plasma IL-6 was the most upregulated inflammatory molecule and was responsive to *Ripk3* deficiency. These findings were confirmed by ELISA at earlier time points (Fig. 4b). Furthermore, as in the kidney, LPS also increased *Il6, Ccl5*, and *Cxcl9* expression in the liver, lung, and heart (Fig. S4). However, while *Il-6* expression was *Ripk3*-deficiency-responsive in the liver and lung (Fig. S4a, b), it was RIPK3-independent in the heart (Fig. S4c). These findings support the hypothesis that RIPK3 may mediate systemic inflammation in LPS-induced CSS, and that IL-6 is a key component of this response.

**Table 2:** Olink plasma proteomics.

Cytokine	CSS-AKI WT vs control (fold-change)	*p* value	CSS-AKI Ripk3-KO vs CSS-AKI WT (fold-change)	*p* value
IL6	8.70	0.000	0.56	0.001
IL17F	6.09	0.005	0.23	0.002
TNNI3	4.80	0.004	0.95	0.742
IL17A	4.26	0.000	0.50	0.001
IL10	2.88	0.000	0.86	0.004
IL1B	2.80	0.008	0.82	0.311
GCG	2.46	0.001	0.59	0.000
CSF2	2.01	0.001	0.90	0.368
CCL3	1.88	0.000	1.06	0.199
CCL5	1.83	0.000	0.91	0.013
GHRL	1.76	0.001	0.74	0.001
TNFRSF12A	1.73	0.000	0.73	0.000
CXCL9	1.68	0.000	0.89	0.003
EDA2R	1.57	0.001	0.69	0.000
ITGB6	1.53	0.032	0.82	0.217
ACVRL1	1.42	0.005	0.71	0.002
FAS	1.40	0.002	0.79	0.004
CCL20	1.39	0.000	0.96	0.152
MATN2	1.37	0.000	0.81	0.025
TNF	1.35	0.027	0.66	0.002
FSTL3	1.28	0.000	0.90	0.000
CLMP	1.27	0.001	0.84	0.003
NTF3	1.26	0.007	1.09	0.073
CXCL1	1.25	0.000	1.04	0.015
CCL2	1.24	0.000	1.03	0.000
DLK1	1.21	0.043	0.65	0.001
ERBB4	1.20	0.003	0.75	0.000

Proteins upregulated at 24 h with a *p* value < 0.05 in CSS-AKI WT vs control are shown; *p* values < 0.05 are marked in red for CSS-AKI WT vs control and in blue for CSS-AKI Ripk3-KO vs CSS-AKI WT.

### RIPK3 from inflammatory bone marrow-derived cells contributes to the cytokine storm and kidney dysfunction in CSS-AKI

RIPK3 from BM-derived cells contributes to early kidney inflammation in FA-AKI and AKI-to-CKD progression after kidney ischemia-reperfusion [17, 18]. Thus, we analyzed the role of RIPK3 in BM-derived cells in BM chimeric mice. WT and *Ripk3*-KO mice were lethally irradiated and reconstituted with BM obtained from either *Ripk3*-KO (KO→WT/KO→KO) or WT donors (WT→WT/WT→KO). After LPS injection, and consistent with prior results obtained in constitutive *Ripk3*-KO mice, KO→KO chimeric mice showed kidney protection compared with WT→WT chimeric mice. Ripk3 deficiency in BM cells (KO→WT mice) also improved kidney function, and the reconstitution of *Ripk3*-KO mice with WT BM cells (WT→KO mice) induced more severe kidney failure (Fig. 4c). Moreover, changes in plasma IL-6 were dependent on RIPK3 expression in BM-derived cells (Fig. 4d).

Regarding cytokine expression, KO→KO chimeric mice showed reduced kidney expression of all cytokines and chemokines compared to WT→WT mice. The largest difference was observed for *Il6* (Fig. 4e). All cytokine and chemokine levels were decreased by *Ripk3* deficiency in BM cells (KO→WT mice) and increased upon reconstitution of *Ripk3*-KO mice with WT BM cells (WT→KO). Finally, *Ripk3* deficiency in BM cells prevented kidney infiltration by macrophages and lymphocytes (Fig. 4f).

These findings suggest that the kidney expression of *Il-6*, *Ccl5, Cxcl1 and Cxcl9* in CSS-AKI is dependent on RIPK3 in BM cells. Indeed, the expression of these inflammatory molecules was positively correlated with the severity of kidney dysfunction, as assessed by plasma creatinine and BUN levels (Supplementary Table 1). These results suggest that RIPK3 from the BM and kidney expression of *Il-6* and *Ccl5* may play a key role in kidney failure during CSS-AKI, while a potential isolated role of *Cxcl1* and *Cxcl9* is less likely, given that downregulation in NLRP3-deficient mice was not associated with kidney protection (Fig. S3).

### RIPK3 mediates IL-6 expression in macrophages but not in tubular cells

**Fig. 5:**
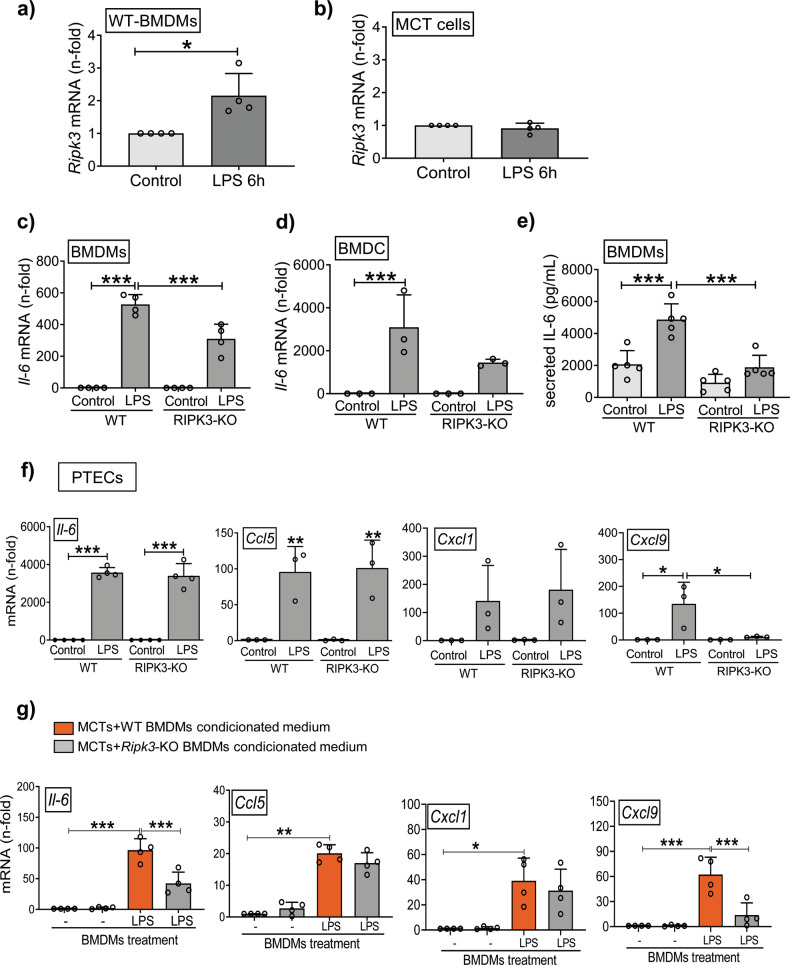
RIPK3 mediates the expression of IL-6 in cultured bone marrow-derived cells, but not in tubular cells. **a**, **b** *Ripk3* mRNA expression was measured in bone marrow-derived macrophages (BMDM) and in tubular cells (MCTs) stimulated with 100 ng/ml LPS for 6 h. **c**, **d** The effect of LPS stimulation (100 ng/mL for 6 h) over *Il-6* mRNA expression was measured by RT-PCR in WT and *Ripk3*-KO BMDMs (**c**) and BM dendritic cells**. e** Secreted IL-6, measured by ELISA in supernatants of WT and *Ripk3*-KO BMDMs stimulated with 100 ng/ml LPS for 6 h. **f** *Il-6, Ccl5, Cxcl1*, and *Cxcl9* mRNA expression in WT and Ripk3-KO primary tubular epithelial cells (PTECs) stimulated with 100 ng/ml LPS for 6 h. **g** MCT cells were cultured with conditioned medium from BMDM, WT, and *Ripk3*-KO, which had been stimulated with 100 ng/mL LPS for 6 h. Tubular cell *Il-6, Ccl5, Cxcl1* and *Cxcl9* mRNA levels were measured by RT-PCR. **a**–**g** Mean ± SD of 4 independent experiments. *p < 0.05; **p < 0.01; ***p < 0.001.

In vitro studies in BM-derived and renal tubular cells were performed to determine the role of RIPK3 in each cell type. LPS increased *Ripk3* expression in BM-derived macrophages from WT mice (WT-BMDMs), but not in murine cortical tubular (MCT) cells (Fig. 5a, b). Moreover, *Il-6* expression and IL-6 secretion were lower in BMDMs from *Ripk3*-KO mice than from WT mice. Similar results were observed in BM dendritic cells (BMDCs) (Fig. 5c–e). These data suggested that BM-derived cells are a key source of IL-6 in CSS-AKI.

However, in primary tubular epithelial cells (PTECs), *Ripk3* deficiency only prevented *Cxcl9* expression, with no effect on *Il-6, Ccl5*, and *Cxcl1* expression (Fig. 5f). Since intracellular RIPK3 did not account for the increased tubular cell expression of diverse chemokines in response to LPS, we analyzed whether LPS-stimulated BMDMs promoted proinflammatory responses in tubular cells that may contribute to the amplification of kidney inflammation. To assess the impact of the BMDM secretome on tubular cells, we stimulated MCT cells with conditioned medium from BMDM-WT or BMDM-*Ripk3*-KO mice, stimulated or not stimulated with LPS. Conditioned medium from LPS-stimulated BMDM-WT induced the expression of all inflammatory cytokines and chemokines in tubular cells. Additionally, conditioned media from BMDM-*Ripk3*-KO mice did not increase *Il-6* and *Cxcl9* expression, whereas *Ccl5* and *Cxcl1* barely changed (Fig. 5g). These results support the concept that RIPK3 expression by BM-derived cells contributes to kidney inflammation in CSS-AKI by promoting the recruitment of secondary proinflammatory responses in tubular cells.

### The RIPK3-IL-6 axis plays a key role in cytokine storm and kidney dysfunction in CSS-AKI

**Fig. 6:**
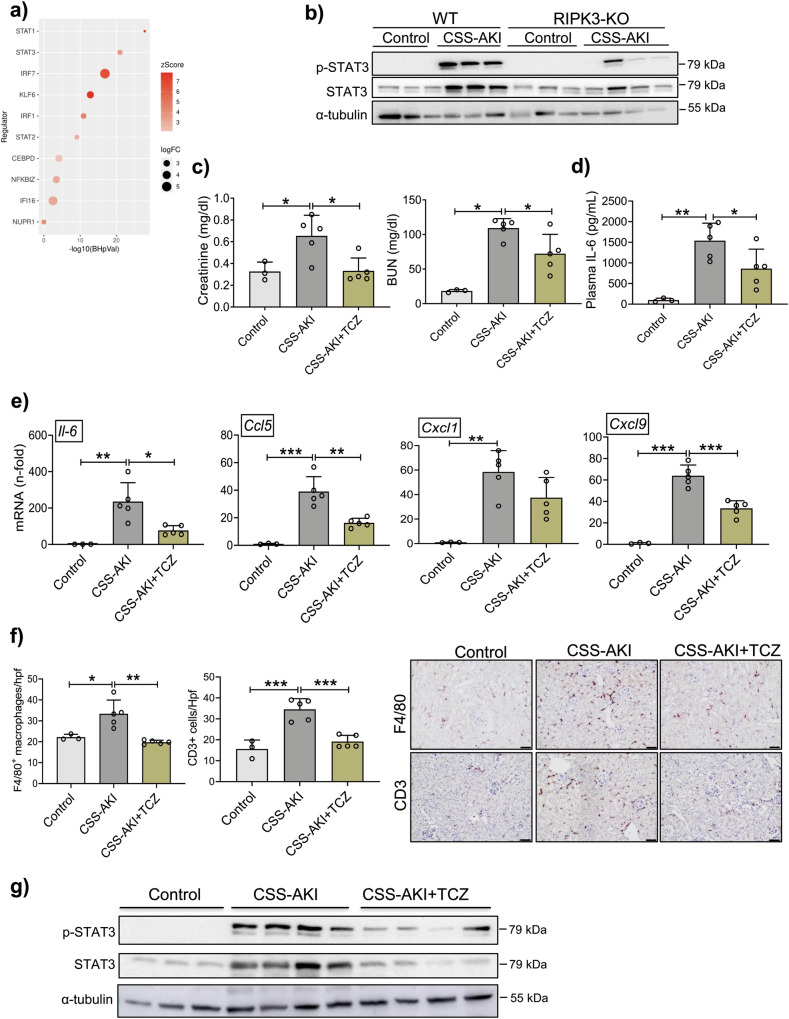
Targeting the IL-6 receptor (IL-6R) improves renal function and reduces kidney inflammation during CSS-AKI. **a** Bubble plot of the top 10 transcriptional regulators that were both overexpressed [Log fold change (FC) > 2.0] and overactivated (*z*-score > 2.0) in the kidneys of mice with CSS-AKI versus control. The *x*-axis represents the statistical significance of transcriptional regulator enrichments expressed as −log10(BHpVa). The bubble color represents the activity of regulators expressed as the *z*-score values calculated by IPA. The size of dots represents the log FC between CSS-AKI and Control. **b** Western blot of p-STAT3 and total STAT3 in WT and RIPK3-KO mice control or with CSS-AKI at 24 h after LPS injection. Representative Western blot of 3–4 mice per group. **c**–**g** WT mice were treated with humanized anti-IL6R antibody Tocilizumab (TCZ, 10 mg/kg) at the time of CSS-AKI induction**. c** Renal function assessed by plasma creatinine and BUN levels at 24 h after CSS-AKI. **d** Plasma levels of IL-6 measured by ELISA at 24 h. **e** Kidney mRNA levels of proinflammatory cytokines measured by RT-PCR at 24 h of CSS-AKI induction. **f** Kidney interstitial infiltration by F4/80 positive interstitial macrophages and CD3+ T cells in CSS-AKI at 24 h. Representative images (Magnification 200×) and quantification. Scale 50 µm. **c**–**f** Mean ± SD of 3–5 mice per group. *p < 0.05; **p < 0.01; ***p < 0.001. **g** Western blot at 24 h after injury of p-STAT3 and total STAT3 in control kidneys and kidneys with CSS-AKI treated or not with TCZ. Representative Western blot of 3–4 mice per group.

After excluding necroptosis and the NLRP3 inflammasome as key contributors to protection from LPS-induced cytokine storm and CSS-AKI induced by *Ripk3* deficiency, we explored the role of IL-6 since: (a) it was the most increased inflammatory mediator in the kidneys and plasma of mice with CSS-AKI, (b) it was also the most responsive to *Ripk3* deficiency, including in BM-selective *Ripk3* deficiency, and (c) its kidney expression was RIPK3-dependent and positively correlated with the severity of kidney failure (Figs. 1d–f, 2a and 4a, b, e). In line with this, kidney RNA-seq comparing CSS-AKI and control mice revealed that STAT3, a transcription factor downstream of IL-6, was among the top 10 most upregulated (Log FC > 2.0) and activated (*z*-score > 2) transcription factors in CSS-AKI compared to control kidneys and was the second most significantly enriched (Fig. 6a). In this regard, kidney STAT3 expression and phosphorylation were reduced in *Ripk3* deficient mice with CSS-AKI compared with WT mice, as observed by western blotting (Fig. 6b), which is consistent with the negative impact of *Ripk3* deficiency on IL-6 signaling and STAT-3 activation.

To further characterize the role of IL-6 in mediating the deleterious effect of RIPK3 in CSS-AKI, the IL-6 receptor-targeting antibody, tocilizumab, was used. Tocilizumab improved kidney function and reduced plasma IL-6 levels, kidney expression of BM RIPK3-dependent cytokines (*Il-6*, *Ccl5, Cxcl1, Cxcl9*), and leukocyte infiltration (Fig. 6c–f). Moreover, tocilizumab reduced the expression and phosphorylation of STAT3 in CSS-AKI mice (Fig. 6g). These data corroborate that the RIPK3-IL-6 axis plays a key role in CSS-AKI development.

### Clinical relevance: evidence for RIPK3-IL-6 axis activation in human CSS-AKI as exemplified by COVID-19

COVID-19 is a cause of CSS-AKI, which has been well characterized at the kidney and molecular levels, in which IL-6 plays a pathogenic role, as demonstrated by the kidney-protective effect of tocilizumab in clinical trials [19–21]. Data from a kidney transcriptomics dataset of nine COVID-19 AKI patients and five control samples identified a significant upregulation of both *RIPK3* and *IL6* expression, consistent with the activation of the RIPK3-IL-6 pathway in human CSS-AKI [21] (Fig. S5).

## Discussion

The main finding was that RIPK3 contributes to systemic inflammation, AKI, and mortality during CSS. Specifically, RIPK3 from BM mediates the increase in plasma IL-6 and other inflammatory mediators, leading to injury and inflammation in different organs, including the kidneys (Fig. S6). Overall, the results point to a novel necroptosis-independent and NLRP3 inflammasome-independent RIPK3-IL-6 axis that contributes to CSS severity. This information may be clinically relevant since anti-IL-6 therapies are currently in use for CSS (https://www.ema.europa.eu/en/documents/product-information/roactemra-epar-product-information_en.pdf). The observation that targeting an upstream regulator of IL-6 within BM cells may also protect against CSS opens the door to exploring alternative therapeutic approaches that target a limited cell set at an earlier point in the pathogenic cascade.

CSS is characterized by an exacerbated inflammatory response that may lead to multiorgan failure, including AKI and death [4]. CSS may be caused by sepsis, viral infection, and sterile inflammation triggered by treatments, such as CAR-T therapy [6–10]. In this context, SARS-CoV-2 infection, which induces CSS, is associated with a high incidence of AKI and may be treated with tocilizumab, similar to CAR-T cell-associated severe or life-threatening cytokine release syndrome (https://www.ema.europa.eu/en/documents/product-information/roactemra-epar-product-information_en.pdf). Therefore, although CSS is mostly associated with sepsis, it may occur in other pathological conditions relevant to kidney disease, including aseptic conditions. Here, we studied sterile CSS caused by the injection of LPS, also known as endotoxin, a membrane component of gram-negative bacteria that promotes both systemic and local synthesis and secretion of proinflammatory cytokines. Endotoxins may be present in sterilized products, such as medical devices [22]. LPS administration does not exactly reproduce human sepsis, in which live bacteria usually proliferate in the bloodstream, but it is a good model of sterile CSS, since it induces a strong systemic inflammatory response affecting different organs such as the kidneys, liver, and lungs, and allows the dissociation of inflammation from antibacterial responses [23].

RIPK3 is a key necroptosis pathway protein that is activated by RIPK1 and phosphorylates MLKL, the necroptosis executor. Targeting RIPK3 prevented necroptosis activation and improved renal function in ischemia/reperfusion-induced AKI, in late time points of folic acid-AKI, cisplatin-AKI, and oxalate nephropathy [24–27]. However, RIPK3 can also modulate kidney inflammation independently of necroptosis activation, as observed in CLP-sepsis-AKI and in early inflammation in folic acid-AKI [16, 17]. Consistent with these reports, we observed that RIPK3 deficiency reduces kidney inflammation and improves renal function and survival in murine CSS induced by LPS injection. However, treatment with the necroptosis inhibitor Nec-1 was not protective, suggesting that the inflammatory effect of RIPK3 is independent of necroptosis in CSS-AKI. Indeed, the sublethal dose of LPS used did not induce tubular cell death as assessed by TUNEL [28], supporting that tubular cell death is not key to kidney injury in this model [29]. In a more severe model of AKI induced by a 2-fold higher LPS dose, GSK´872, an inhibitor of RIPK3, was protective and prevented tubular cell apoptosis, which was observed in untreated mice [30].

Despite observing kidney protection in *Ripk3*-deficient mice with CSS-AKI, the kidney expression of RIPK3 was lower than that of nephrotoxic AKI [17]. This suggests that kidney RIPK3 may not be a key factor in CSS-AKI. In this regard, the results were consistent with the key role of RIPK3 in BM-cells in CSS-AKI, since *Ripk3* deficiency in BM was sufficient to improve renal function and decrease kidney IL-6 and inflammatory infiltrates. Furthermore, targeting RIPK3 did not reduce LPS-induced IL-6 expression in cultured tubular cells, but had an anti-inflammatory effect in cultured macrophages and dendritic cells. Moreover, RIPK3 deficiency also prevented the inflammatory response in other organs, such as the liver, lungs, and heart, supporting the role of RIPK3 in systemic inflammation in CSS. The key role of RIPK3 in BM cells is consistent with previous data on liver fibrosis, nephrotoxic AKI, lung injury, arthritis, and colitis [13, 15, 31–34].

Mechanistically, we tested the role of the NLRP3 inflammasome, as some reports have shown that RIPK3 activates the NLRP3 inflammasome [15, 32], and indeed, *Ripk3* deficiency partially reduced kidney inflammasome activation. However, *Nlrp3* deficiency was not protective in LPS-AKI, suggesting that RIPK3-induced kidney inflammation is inflammasome independent. Similar results were observed in nephrotoxic AKI, where *Ripk3* deficiency reduced kidney inflammasome activation, but *Nlrp3* deficiency did not prevent kidney inflammation [17]. In contrast, IL-6 and IL-6 signaling appeared to be key RIPK3 targets in different locations, as assessed using different methods. IL-6 is a key cytokine in CSS. The COVID-19 pandemic has highlighted its role as a CSS trigger during the SARS-CoV-2 infection [35, 36]. In this regard, targeting IL-6R with tocilizumab protects against CSS-AKI. While some in vitro studies did not show inhibition of murine IL-6 signaling by tocilizumab, these experiments used IL-6 concentrations 50-fold higher than those found in the circulation in our model, and maximum tocilizumab concentrations were 3-fold lower than those expected for the volume of distribution of tocilizumab in mice [37]. In this regard, protection by tocilizumab has been previously reported in several murine models [38–41].

Taken together, these data are consistent with the pathogenic role of RIPK3 in BM-derived cells in CSS, in which RIPK3 drives IL-6 expression, which promotes the activation of STAT3 signaling in the kidney, thereby contributing to the pathogenesis of CSS-AKI (Fig. S6). This observation may lead to the development of novel therapies for CSS that specifically target BM cells. These approaches could reduce the likelihood of potential adverse effects caused by targeting RIPK3 in other cell types. Additionally, RIPK3 acts upstream of IL-6 production, at an earlier point in the pathogenic cascade, which may enhance therapeutic efficacy. The upregulation of other inflammatory mediators (Ccl5, Cxcl1, and Cxcl9) was also RIPK3-dependent, but their induction followed a slower time course than that for IL-6, suggesting that they may amplify the initial CSS response rather than act as key initial triggers of IL-6. In this regard, *Cxcl1* and *Cxcl9* upregulation appears to be NLRP3-dependent, and their downregulation in NLRP3-deficient mice was not associated with improved kidney outcomes.

Although RIPK3 from BM was shown to contribute to CSS, specific BM-derived cell types were not characterized. In addition, while human CSS-AKI data support activation of the RIPK3–IL-6 pathway, clinical translation of these preclinical findings requires validation in interventional studies. Nevertheless, the data presented here offer valuable insights into the pathogenic mechanisms involved.

In conclusion, these data support the role of RIPK3 in BM-derived cells in the induction of systemic inflammation during CSS and identify a novel RIPK3-IL-6 axis that may contribute to organ injury, including the kidneys, following CSS. Therefore, our results suggest that targeting the RIPK3-IL-6 axis in BM-derived cells could be a promising therapeutic tool to mitigate inflammation and tissue injury in CSS.

## Materials and methods

### Animal models

All procedures were conducted in accordance with the NIH Guide for the Care and Use of Laboratory Animals and were approved by the Animal Ethics Committee of the IIS-FJD and Madrid Community (PROEX 070/17, PROEX 64.5/20, PROEX 56.5/23). C57BL/6 female mice (Charles River Laboratories, Ecully, France), 12- to 14-week-old, weighing 19–21 g, were used. Mice were housed in cages at 25 ± 1 °C on a 12-h light/dark cycle and provided free access to food and water. Plasma samples were collected at the time of euthanasia. The kidneys were perfused in situ with cold saline prior to removal. Samples from different tissues (heart, lung, liver) and one kidney were snap-frozen in liquid nitrogen for RNA and protein studies. The remaining tissue samples, including the other kidney, were formalin-fixed and paraffin-embedded for histological studies [42]. Plasma creatinine and BUN levels were measured using the enzymatic Creatinine Plus Version 2 reagent and the UREAL kit, respectively (Roche Diagnostics, Rotkreuz, Switzerland) on a Cobas Integra 400 analyzer (Roche Diagnostics).

LPS-induced endotoxemia is characterized by a systemic cytokine storm and interstitial leukocyte infiltration in the kidney, leading to kidney injury and inflammation. Female wild type (WT) and *Ripk3*-KO mice on the C57BL/6 background (kindly provided by Kim Newton and Vishva Dixit, Genentech, San Francisco, CA, USA) [43] were used in this study. The sample size for the animal models was calculated using the application provided by the Institutional Animal Care and Use Committee (University of Illinois), which predicts the sample size required to achieve statistical significance based on a pilot study. Mice (12- to 14-week-old, 4–8 per experimental group) received an intraperitoneal (i.p.) injection of 5 mg/kg bacterial lipopolysaccharide (LPS, Escherichia coli O111:B4, Sigma-Aldrich, St. Louis, MO, USA), or its vehicle (saline), and were euthanized 1 h, 4 h, or 24 h post-LPS injection. For survival experiments, mice received 15 mg/kg LPS. The dose of LPS was based on previous reports [44].

Folic acid nephropathy is a classical model of kidney tubulointerstitial injury and inflammation characterized by acute renal failure, tubular cell death, interstitial leukocyte infiltration, and subsequent tubular regeneration [17] that has been reported in humans [45]. Mice received a single intraperitoneal injection of 250 mg/kg folic acid (Sigma, St. Louis, MO) in 0.3 mol/l sodium bicarbonate or vehicle and were euthanized at 48 h post-injection.

The necroptosis inhibitor Necrostatin-1 (Nec-1, 1.65 mg/kg, Santa Cruz Biotechnology, Santa Cruz, CA, USA) or DMSO (vehicle) was administered i.p. 30 min prior to LPS injection in WT C57BL/6 mice (Charles River Laboratories, Ecully, France), based on previously reported in the literature [24]. To test the role of the NLRP3 inflammasome, we used *Nlrp3*-KO mice (kindly provided by Pablo Pelegrin, Instituto Murciano De Investigación Biosanitaria, Murcia, Spain). In these experiments, mice were euthanized 24 h after LPS injection (*n* = 3–6 mice per group).

To evaluate the effect of IL-6 pathway inhibition, mice were injected with 10 mg/kg tocilizumab (Actemra®, Roche, Basilea, Switzerland), a humanized monoclonal antibody acting as an IL-6 receptor antagonist, at the time of LPS injection [41, 46].

To generate chimeric mice, WT and Ripk3-KO receptor mice were irradiated with 4.5 Gy in 2 consecutive days to deplete the autologous BM. BM was extracted from the femur and tibia of donor WT or *Ripk3*-KO mice, and 10^7^ cells were transferred to irradiated receptor mice by intravenous injection. After 1 month, CSS-AKI was induced by administering LPS, and mice were euthanized 24 h later. The efficacy of BM reconstitution was tested by PCR analysis on blood samples with the same primers used for tail Ripk3 genotyping [17].

### RNA extraction and real-time polymerase chain reaction

Total RNA was extracted by the TRI Reagent method (Invitrogen-Thermo Fisher Scientific, Waltham, MA, USA), and 1 µg was reverse transcribed with High-Capacity cDNA Archive Kit (Applied Biosystems, Foster City, CA, USA). Quantitative PCR was performed in a Quant Studio 5 PCR System with the QuantStudio 3/5 Real-Time PCR Software using predeveloped primers (Applied Biosystems), and RNA expression of different genes was corrected for GAPDH expression [47].

### Histological assessment of tubular injury

Kidneys from mice were fixed in formalin for at least 48 h and embedded in paraffin blocks. Sections 3 µm thick were stained with hematoxylin and eosin (H&E) and Periodic Acid-Schiff (PAS) using standard histological procedures. Tubular damage and inflammation were evaluated in four randomly chosen fields (two cortical and two from the outer stripe of the outer medulla, OSOM) at 400× magnification on H&E-stained sections. PAS staining was performed to enhance lesion visualization and confirm brush border loss and intratubular cast formation in renal tissue sections. Tubular injury was defined by the presence of lumen dilation and/or epithelial attenuation, cytoplasmic vacuolization or hydropic degeneration, cast formation, and epithelial regeneration (mitotic figures). Inflammation was assessed based on the presence of inflammatory cells infiltrating the renal interstitium. Each histological parameter was graded on a scale from 0 to 4: 0 = none, 1 = mild, 2 = moderate, 3 = marked, and 4 = severe, based on both the intensity and extent of the lesion (0%, ≤10%, 11–25%, 26–50%, >50%, respectively). The final renal injury score for each mouse was determined by summing the scores of all histological parameters within each individual field, followed by calculating the average of these sums across the four fields analyzed per animal. The scale for final score is 0–5 = mild, 6–12 = moderate, 13–18 = marked. Samples were examined in a blind manner.

### Immunohistochemistry

Kidney tissue staining was performed in 3 µm thick sections of paraffin-embedded tissue using a PT-link device with a low pH solution. Sections were then washed with PBS for 5 min, blocked by incubation with 4% PBS/BSA + 6% serum, and incubated with primary antibody overnight. Primary antibodies were rabbit polyclonal anti-F4/80 (1:100, Ref. 70076, CST, Danvers, MA, USA) and anti-CD3 (ready to use, Ref. M7254, Agilent-DAKO, Santa Clara, CA, USA). Sections were counterstained with Carazzi’s hematoxylin. Negative controls included incubation with a non-specific immunoglobulin of the same isotype as the primary antibody. The total number of positive cells was quantitated in 20 randomly chosen fields [20×] using Image-Pro Plus software (Media Cybernetics, Silver Spring, MD, USA). Samples were examined in a blind manner.

### Western blot

Tissue samples were homogenized in lysis buffer T-PER Tissue Protein Extraction Reagent (Thermo Fisher Scientific), then separated by 10% SDS-PAGE under reducing conditions. After electrophoresis, samples were transferred to PVDF membranes (Millipore-Merck, Darmstadt, Germany), blocked with 5% skimmed milk in PBS/0.5% v/v Tween 20 for 1 h, washed with PBS/Tween, and incubated with anti-RIPK3 (1:1000, Ref. 2283, ProSci, Poway, CA, USA), anti-cleaved-caspase 1 (1:500, Ref. AG20B-0042-C100, Adipogen, San Diego, CA), anti-NLRP3 (1:500, Ref. D4D8T, CST), anti-phospho-Stat3 (1:1000, CST) and Stat3 (1:1000, CST) antibodies diluted in 5% milk PBS/Tween. Blots were washed with PBS/Tween, incubated with appropriate horseradish peroxidase-conjugated secondary antibody (1:5000, GEHealthcare, Aylesbury, UK), developed with the chemiluminescence method (ECL) (Thermo Fisher Scientific), and probed with mouse monoclonal anti-α-tubulin (1:10000, Ref. T5168, Sigma-Aldrich) or anti-GAPDH (1:5000, Ref. mab374, Millipore-Merck) antibodies. Levels of expression were corrected for minor differences in loading. *Full and uncropped gel scans merged with molecular weight images for all presented western blots are shown as “Original Data”*.

### Immunofluorescence

RIPK3 immunofluorescence in tissue sections was performed using anti-RIPK3 antibody (1:200, Ref. 2283, ProSci) and the Tyramide SuperBoost™ Kits with Alexa Fluor™ Tyramides, as recommended by the manufacturer (Invitrogen). For macrophage colocalization, RIPK3 staining was followed by incubation overnight at 4 °C with anti-F4/80 (1:50, Ref. MCA497, BioRad, Hercules, CA). Then, slides were washed with PBS and incubated with anti-rat Alexa 633 conjugated secondary antibodies (1:200, Invitrogen). Nuclei were counterstained with DAPI (Sigma-Aldrich). Colocalization images were obtained using confocal fluorescence microscopy (LEICA TCS SP5 II) and imaging software (“LAS AF,” Leica, Wetzlar, Germany).

### ELISA

Plasma IL-6 (BD Pharmingen, San Diego, CA) was assessed by ELISA according to the manufacturer’s instructions.

### RNAseq

The study was performed at Unidad Genómica Cantoblanco, Fundación Parque Científico de Madrid, Madrid, Spain. Total RNA was extracted with PureLink RNA Mini Kit (Invitrogen, Thermo Fisher, Waltham, MA) from control, and CSS-AKI (3 animals per group) obtained 24 h post-induction of CSS-AKI. From 1 μg total kidney RNA, the PolyA+ fraction was purified and randomly fragmented, converted to double-stranded cDNA, and processed through subsequent enzymatic treatments of end-repair, dA-tailing, and ligation to adapters with NEBNext® Ultra™ II DNA Library Prep Kit (New England Biolabs, Ipswich, MA) following the manufacturer’s recommendations. The adapter-ligated library was completed by PCR with Illumina PE primers (10 cycles). The resulting purified cDNA library was applied to an Illumina flow cell for cluster generation and sequenced for 50 bases in a single-read format (Illumina HiSeq 2000). Sequencing quality was first checked with FastQC. The FASTQ files were pre-processed to filter by size, quality, and content of Ns using the Prinseq tool. Reads were then aligned to the mouse genome (GRCm38.p5. genome) with TopHat-2.0.1050. Transcript assembly, estimation of their abundance, and differential expression were calculated with Cufflinks-Cuffdiff in combination with CummeRbund 26. Transcripts with FPKM expression values lower than 0.05 in both conditions were considered not expressed and were excluded from further analysis. These data have been deposited in the Gene Expression Omnibus (GEO) database (GSE243633). Upstream regulator analyses were performed using Ingenuity Pathway Analysis (IPA, Quiagen, Hilden, Alemania).

### Olink inflammation-related biomarker analysis

Plasma and kidney samples from control (*n* = 3), WT CSS-AKI (*n* = 4), and Ripk3-KO CSS-AKI (*n* = 4) mice were evaluated using the Olink mouse exploratory panel (Olink Proteomics AB, Uppsala, Sweden), which allows for simultaneous analysis of 92 biomarkers. In summary, the target protein binds specifically to the double-oligonucleotide-labeled antibody probe, and the oligonucleotide sequence is quantified via microfluidic real-time PCR amplification (Cobiomic Bioscience S.L, Córdoba, Spain).

### Cells and reagents

MCT cells are a proximal tubular cell line harvested originally from the renal cortex of SJL mice and have been extensively characterized [48]. They were cultured in RPMI 1640 (GIBCO, Grand Island, NY, USA), 10% decomplemented fetal bovine serum (FBS), 2 mM glutamine, 100 U/mL penicillin, and 100 µg/mL streptomycin, in 5% CO_2_ at 37 °C, Haverty [48].

BM cells were collected from the femur and tibia of WT and Ripk3-KO mice, sacrificed with CO_2_. Isolated tibia and femur were flushed with RPMI 1640 (10% FBS). BM cells were cultured for 4 days in RPMI 1640 supplemented with 10% FCS, 2 mM glutamine, 100 U/ml penicillin, and 100 µg/ml streptomycin [17]. To this medium, 5 ng/ml M-CSF (Biolegend, San Diego, CA, USA) was added for BM-derived macrophage (BMDM) cultures, while 5 ng/ml GM-CSF (Biolegend) and 5 ng/ml IL-4 (R&D Systems) were added for BMDC culture.

PTECs were obtained from WT and Ripk3-KO mice [17]. After sacrificing with CO_2_, the kidney cortex was decapsulated, dissected from the medulla, sliced, minced, and digested with 1.25 mg/mL collagenase Type I (GIBCO) at 37 °C for 20 min. The rubber end of a syringe plunger was used to filter the pellet through a 100-μm cell strainer (Falcon-Corning, Corning, NY, USA) over a 50-ml conical tube by washing with Medium 199 1X (GIBCO). After depleting red blood cells with erythrocyte lysis buffer (154 mM NH_4_Cl, 10 mM KHCO_3_, 0.5 M EDTA), the pellet was passed through a 40-µm cell strainer (Falcon) and resuspended in RPMI 1640 10% FBS, 100 U/mL penicillin, 100 μg/mL streptomycin, 2 mM glutamine, and ITS (Thermo Fisher, Waltham, MA, USA). Cells were seeded in gelatin type B-coated plates. After 7 days, cells were reseeded in the corresponding plates for experiments.

Subconfluent cells were rested in serum-free medium overnight, and 100 ng/ml LPS was added for 6 h.

To test the impact of the BMDM secretome on MCT cells, WT and *Ripk3*-KO BMDM were stimulated with 100 ng/mL LPS for 6 h. Then, the media was replaced by fresh media, and the cells were further incubated for 24 h. Then, MCT cells were cultured in BMDM-conditioned media for 24 h [17].

### Data mining

To explore the clinical relevance of the murine findings in CSS-AKI, we chose AKI associated to COVID-19, a recognized cause of CSS-AKI [19] in which targeting IL-6 pathway activation with tocilizumab decreased the risk of early death (rate ratio: 0.85; 95% CI: 0.76-0.94) and of severe AKI requiring kidney replacement therapy (rate ratio: 0.72, 0.58–0.90) [20]. Additionally, the literature on COVID-19 CSS included publicly available human data on kidney tissue transcriptomics representing COVID-19 AKI and control kidneys [21]. We searched in GEO and PubMed using the keywords IL-6 AND RIPK3 AND COVID-19. GSE202182 data were downloaded from https://www.ncbi.nlm.nih.gov/geo/ and processed using the tool GEO2R. Differences between healthy and COVID-19 AKI subjects were assessed by the Mann–Whitney test. An adjusted *p* value < 0.05 was considered statistically significant. IL-6 and RIPK3 were considered genes of interest.

### Statistics

Statistical analyses were performed using GraphPad Prism 8.02 (GraphPad Software, La Jolla, CA, USA). Normal distributions were tested using the Shapiro–Wilk test previously to the statistical analyses. Data from all assays passed tests for normality. Means among three or more groups were compared using ANOVA with Tukey’s post-hoc test, one-way ANOVA for comparison with one independent variable, and two-way ANOVA for comparison with two independent variables. A two-tailed Student’s *t*-test was used for comparisons between two groups. When variances differed significantly, Brown-Forsythe and Welch ANOVA tests were applied. Differences were considered statistically significant at *p* < 0.05. All the experiments were performed at least three times.

## Supplementary information

Supplemental Data

Uncropped Original Western Blot

Reproducibility Checklist

## Data Availability

The data of RNA-seq in CSS-AKI have been deposited in the NCBI GEO database with accession number GSE243633.
