# Supplementary material for: The RIPK3-IL-6 axis mediates kidney injury in cytokine storm syndrome
Source: Cell Death Dis. 2026 Apr 15;17(1):714. doi: 10.1038/s41419-026-08686-1 (PMC13470091; doi:10.1038/s41419-026-08686-1)
Supplement: Supplementary file 1 [file 41419_2026_8686_MOESM1_ESM.pdf]

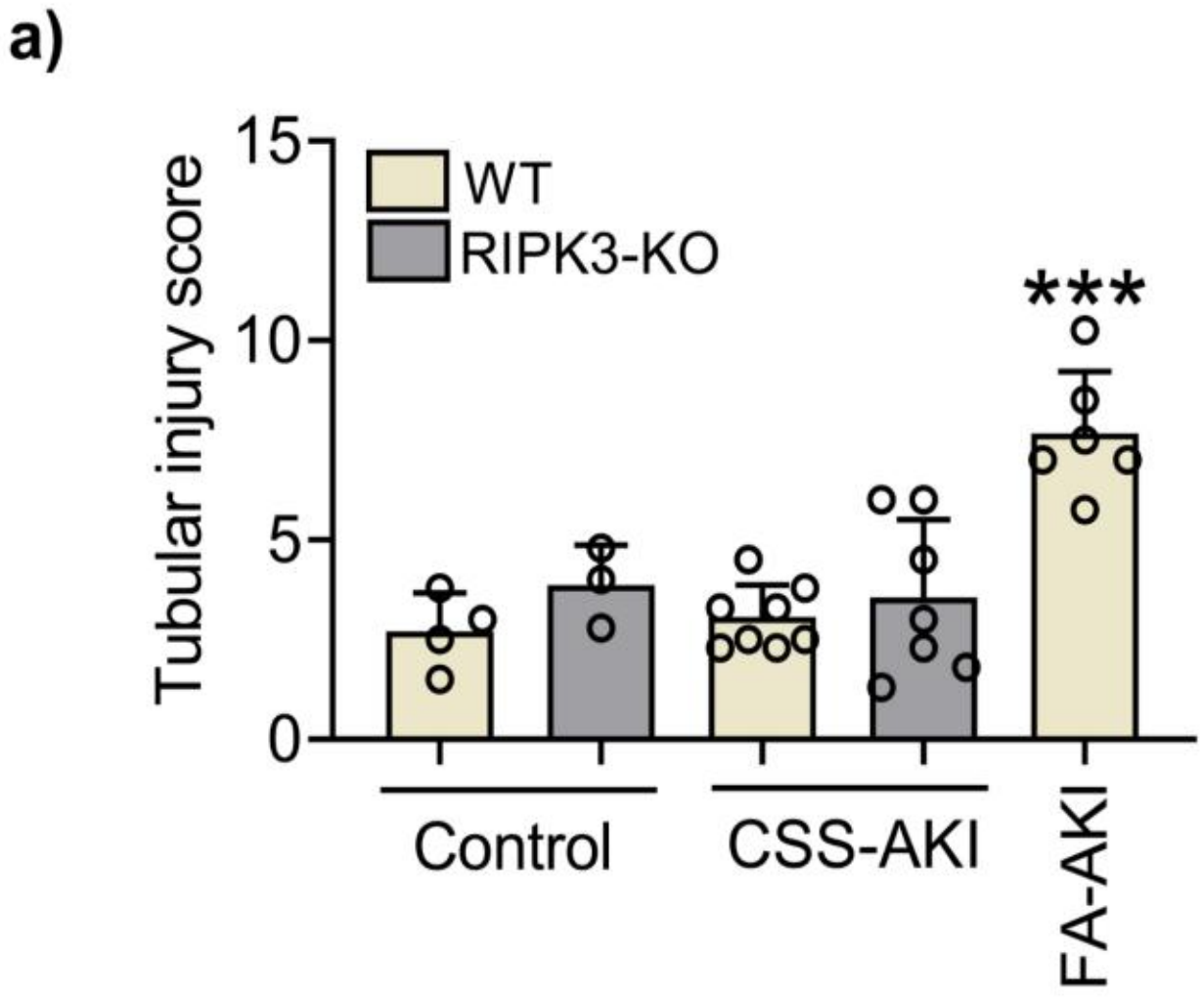

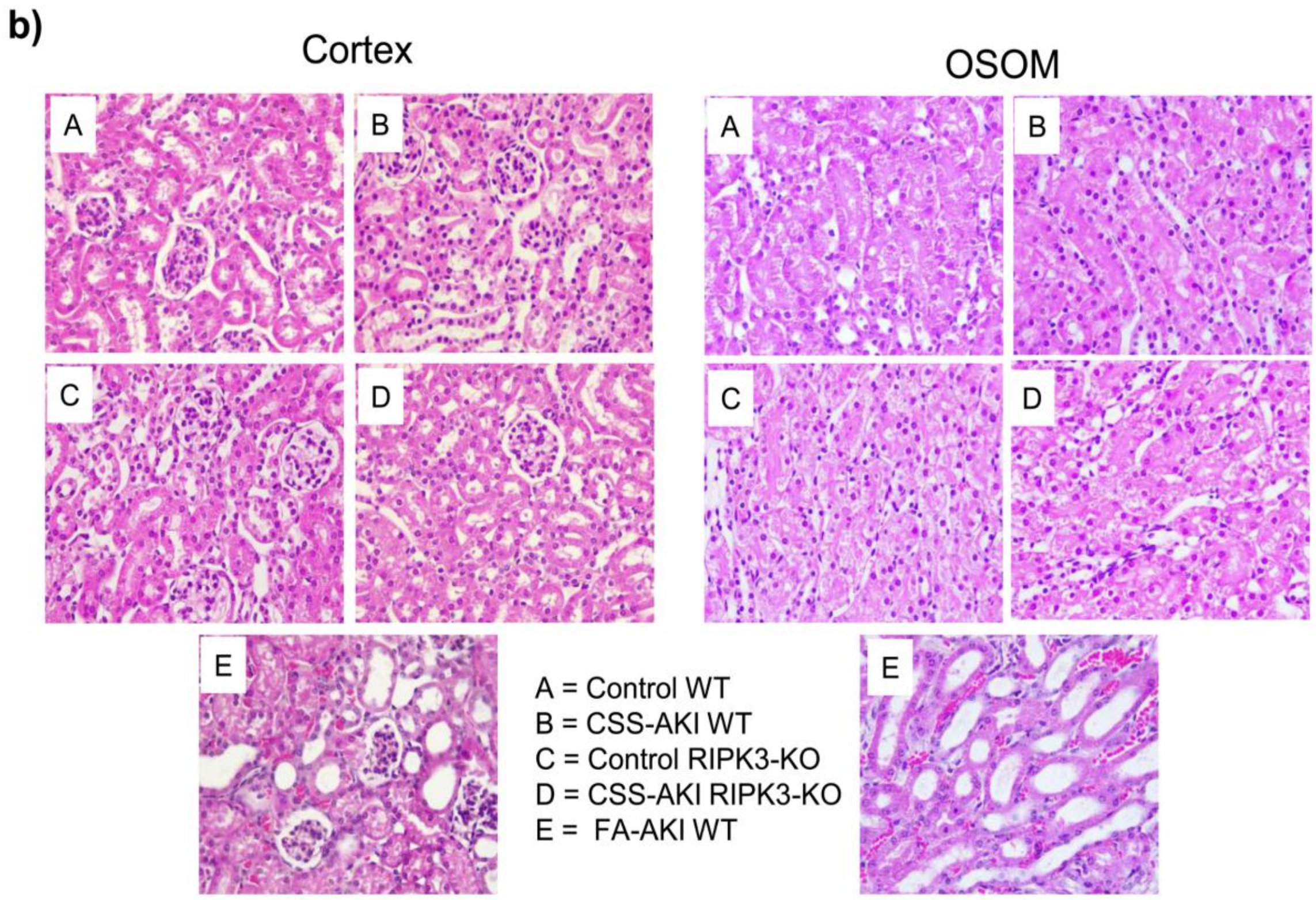

Fig. S1. CSS-AKI at 24 hours develops minor tubular injury in kidneys compared with FA-AKI at 48h. Quantification (**a**) and representative image (**b**) of H&E staining. Magnification 400x. Mean ± SD of 4-8 mice per group. ***p<0.001 vs Control. OSOM: outer stripe of the outer medulla.

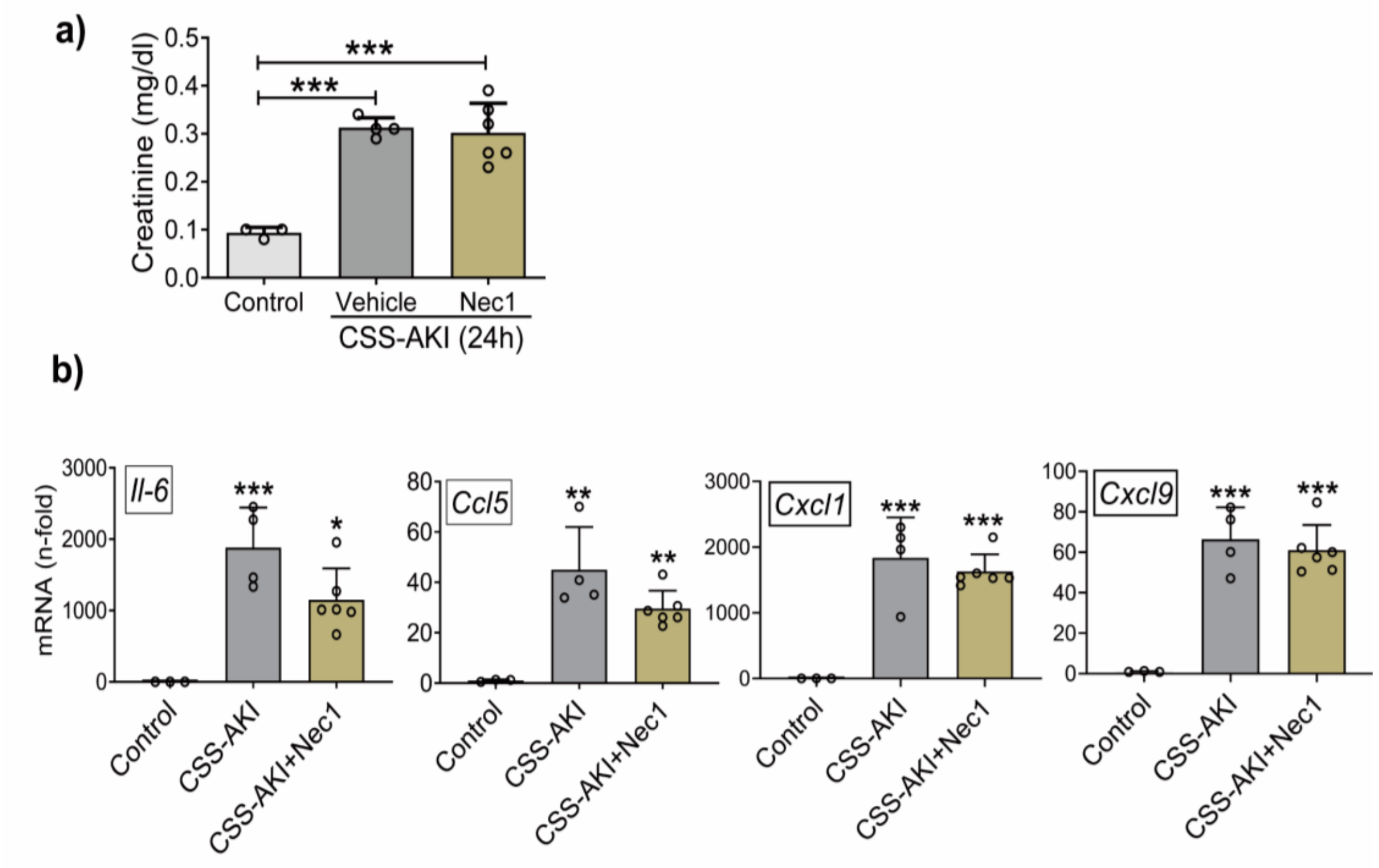

Fig. S2. Necroptosis inhibition does not protect from CSS-AKI kidney failure. WT mice were treated with the necroptosis inhibitor Necrostatin-1 (1.65 mg/kg) 30 minutes before CSS-AKI induction. **a)** Renal function assessed by plasma creatinine levels at 24 hours after CSS-AKI. **b)** Kidney mRNA levels of proinflammatory cytokines measured by RT-PCR 24 hours after induction of CSS-AKI**. a-b)** Mean ± SD of 3-6 mice per group. *p<0.05 vs Control; **p<0.01 vs Control; ***p<0.001 vs Control.

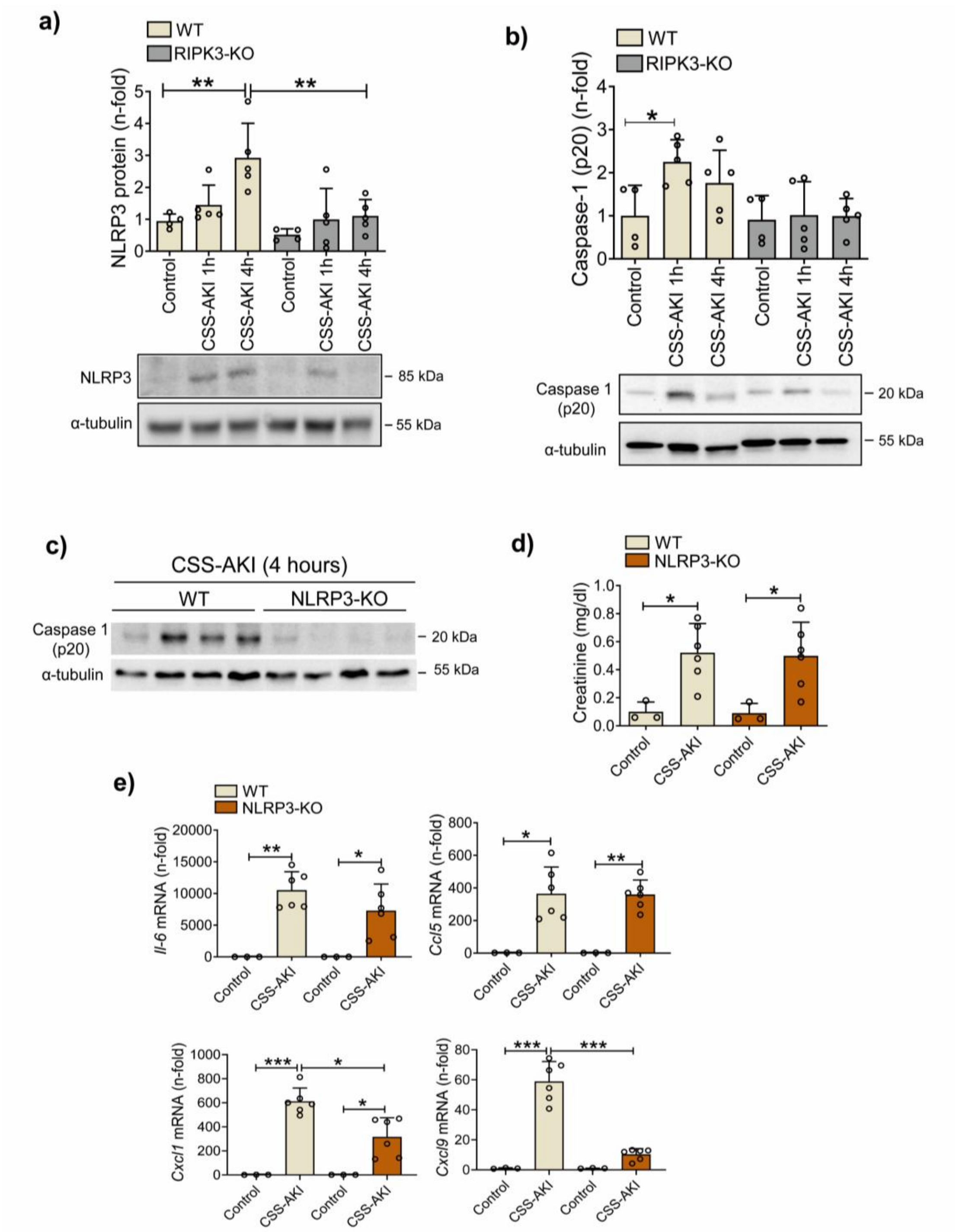

Fig. S3. NLRP3 targeting does not protect from CSS-AKI kidney failure. a, b) Representative western blot and quantification of kidney NLRP3 (a) and cleaved caspase 1 (b) in CSS-AKI at 1 and 4 hours. Mean ± SD of 4-5 animals per group. *p<0.05; **p<0.01. **c)** Western blot of cleaved caspase 1. Cleaved caspase 1 is not observed in Nlrp3-KO mice with CSS-AKI, supporting the NLRP3-dependence of caspase 1 cleavage in this model. Representative western blot. **d)** Kidney function was assessed by plasma creatinine at 24 hours after CSS-AKI induction in WT and

Nlrp3-KO mice. **e)** Kidney Il-6, Ccl5, Cxcl1 and Cxcl9 mRNA level expression assessed by RT-PCR at 24 hours of CSS-AKI. **d, e**) Mean ± SD of 3-6 mice per group. *p<0.05; **p<0.01; ***p<0.001.

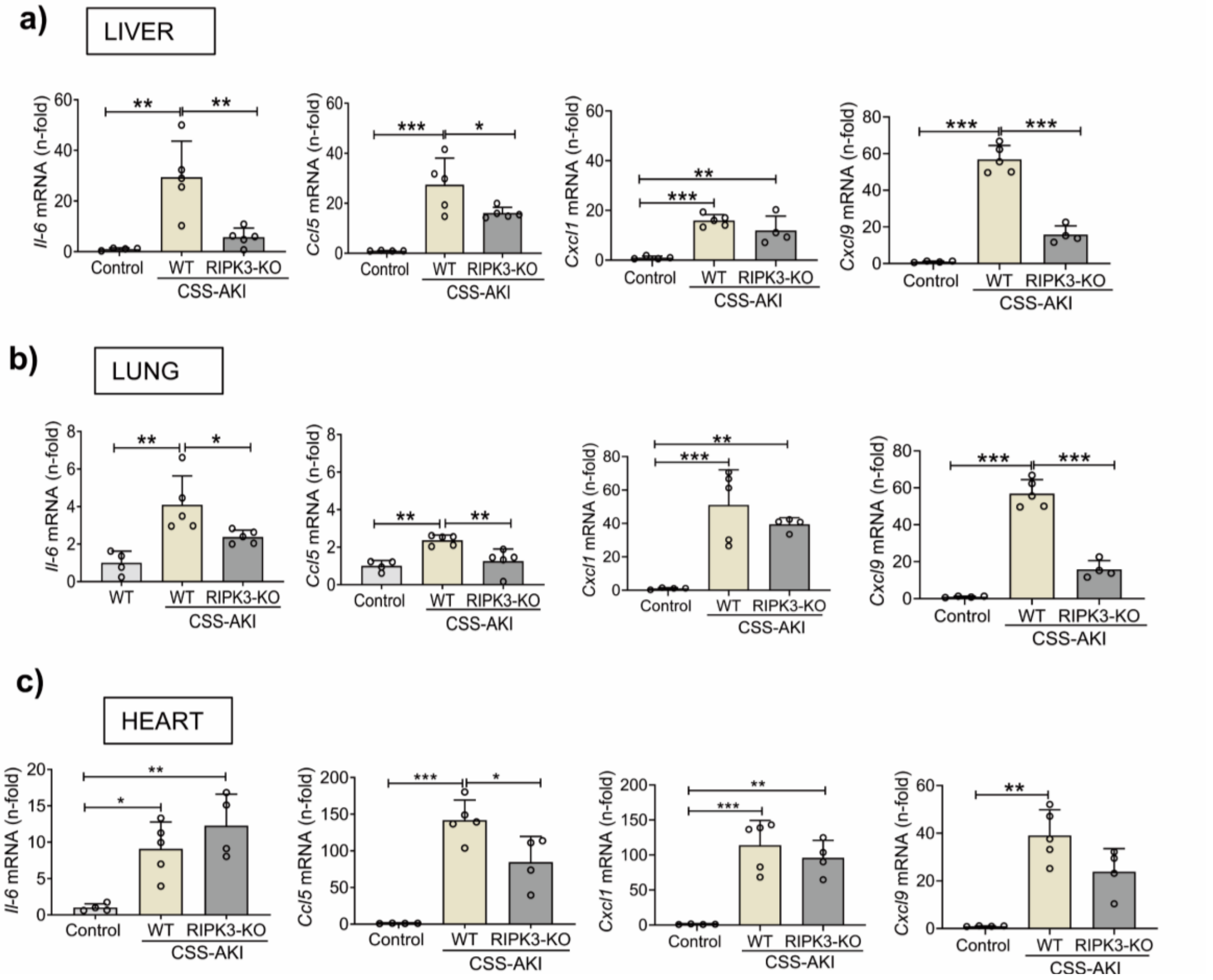

Fig. S4. ***Ripk3* deficiency reduces systemic inflammation in CSS. a-c)** *Il-6, Ccl5, Cxcl1* and *Cxcl9,* mRNA levels, measured by RT-PCR, in liver (a), lung (b) and heart (c) of mice with CSS at 24 hours. Mean ± SD of 4-5 animals per group. *p<0.05; **p<0.01; ***p<0.001.

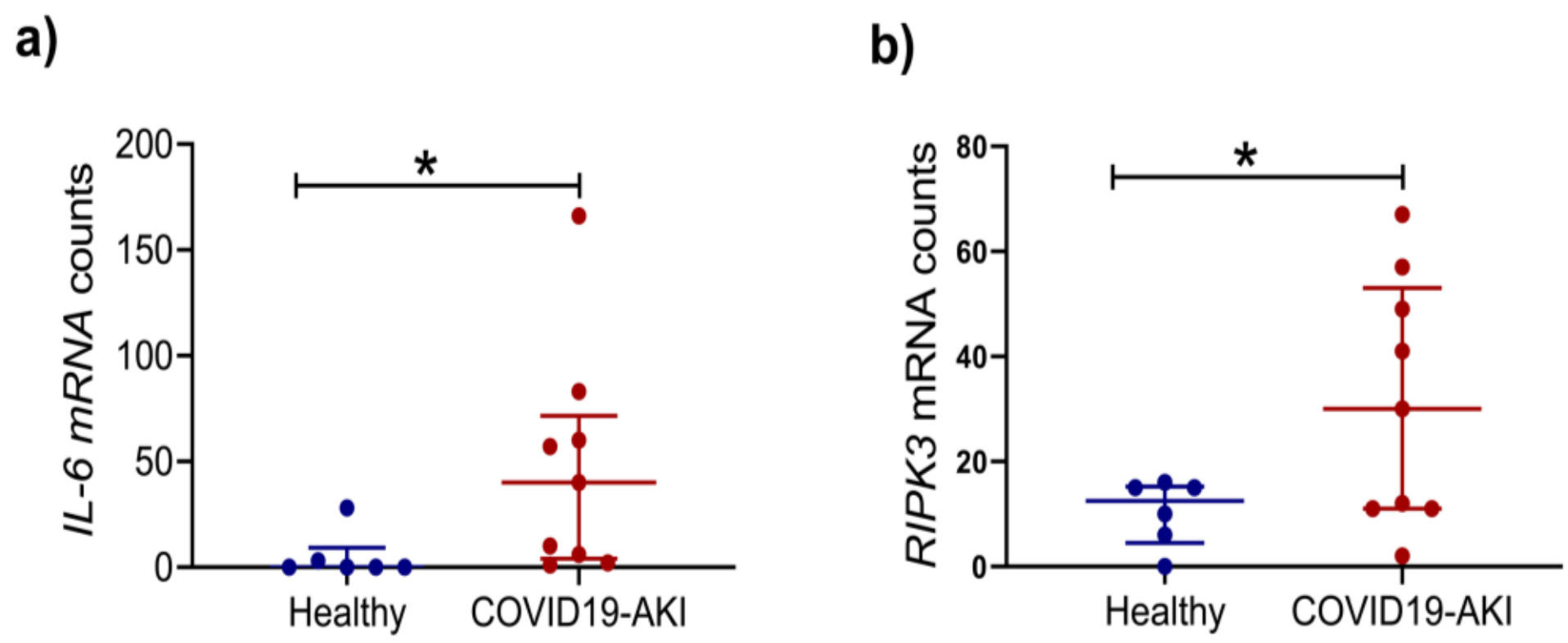

Fig. S5. Human kidney transcriptomics in CSS-AKI. Datamining of a human kidney transcriptomics dataset representing AKI associated to COVID-19 CSS and controls (GSE202182[6]) identified upregulation of *IL-6* (**a**) and *RIPK3* (**b**) mRNA in AKI associated to COVID-19 CSS. Data expressed as median with interquartile range of 6-9 subjects per group. *p <0.05

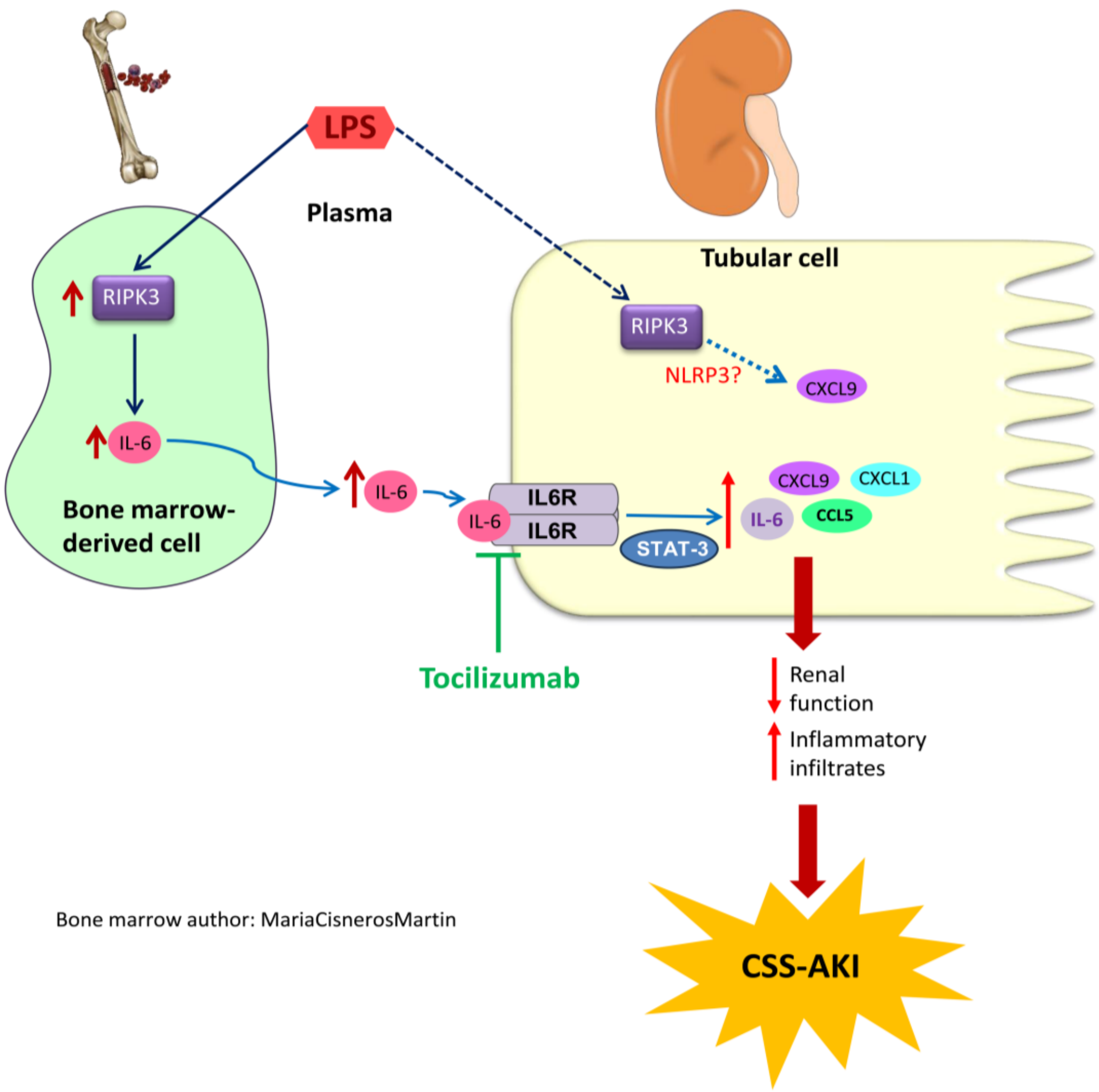

Fig. S6. Conceptual representation of RIPK3 role in CSS-AKI. Cytokine storm syndrome (CSS) is defined as a massive immune response that can lead to multi-organ dysfunction, including AKI, and even death. *In vivo* and *in vitro* studies have shown that RIPK3 in bone marrow cells promotes the expression of IL-6, thereby increasing plasma levels of this cytokine. Plasmatic IL-6 then reaches the kidney, where it activates the STAT3 pathway and triggers the expression of renal IL-6, CCL5, CXCL1 and CXCL9 leading to inflammatory infiltrates and renal injury. Targeting IL-6R inhibits IL-6 signaling in the kidney, preventing cytokine expression, inflammatory infiltrate and kidney dysfunction. In parallel, RIPK3 also induced the kidney expression of CXCL9, maybe through NLRP3 inflammasome, but our results do not support a major contribution of NLRP3, at least at the time points analyze

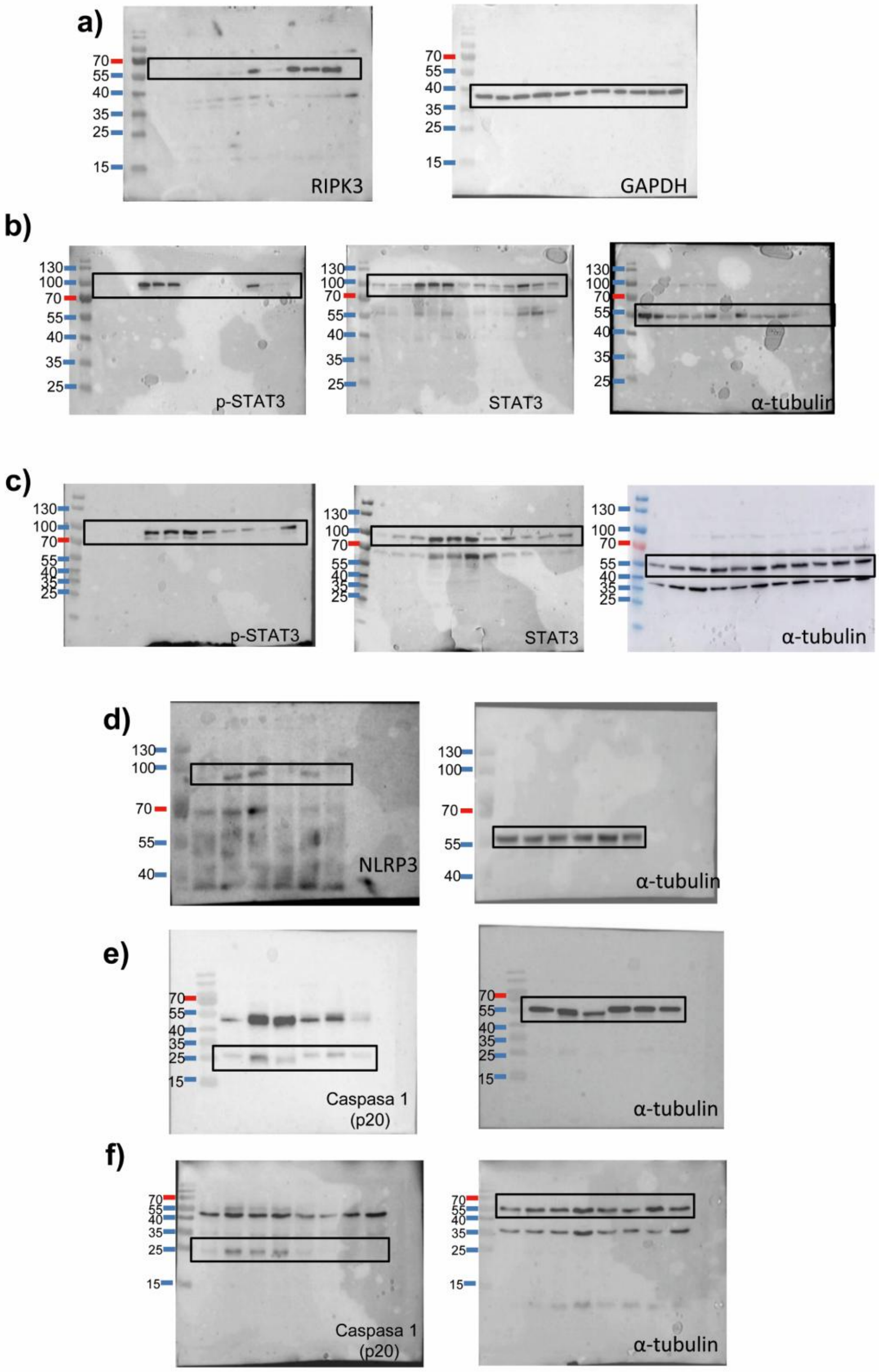

Fig. S7. Uncropped gel scans merged with molecular weight images for all presented western blots. A) Corresponds to Fig. 3.B. B) Corresponds to Fig. 6.B. C) Corresponds to Fig. 6.G. D) Corresponds to SF. 3.A. E) Corresponds to SF. 3.B. F) Corresponds to SF. 3.C

Supplementary Table 1. Correlation between kidney cytokine expression and severity of kidney failure in mouse chimera. Pearson correlation. R values shown.

	**Creatinine**	**BUN**
Il-6	0.630***	0.740***
Ccl5	0.451*	0.630***
Cxcl1	0.473*	0.443*
Cxcl9	0.358	0.439*

***p<0.001; **p<0.01
