# Supplementary material for: The RIPK3-IL-6 axis mediates kidney injury in cytokine storm syndrome
Source: Cell Death Dis. 2026 Apr 15;17(1):714. doi: 10.1038/s41419-026-08686-1 (PMC13470091; doi:10.1038/s41419-026-08686-1)
Supplement: Supplementary file 2 [file 41419_2026_8686_MOESM2_ESM.pdf]

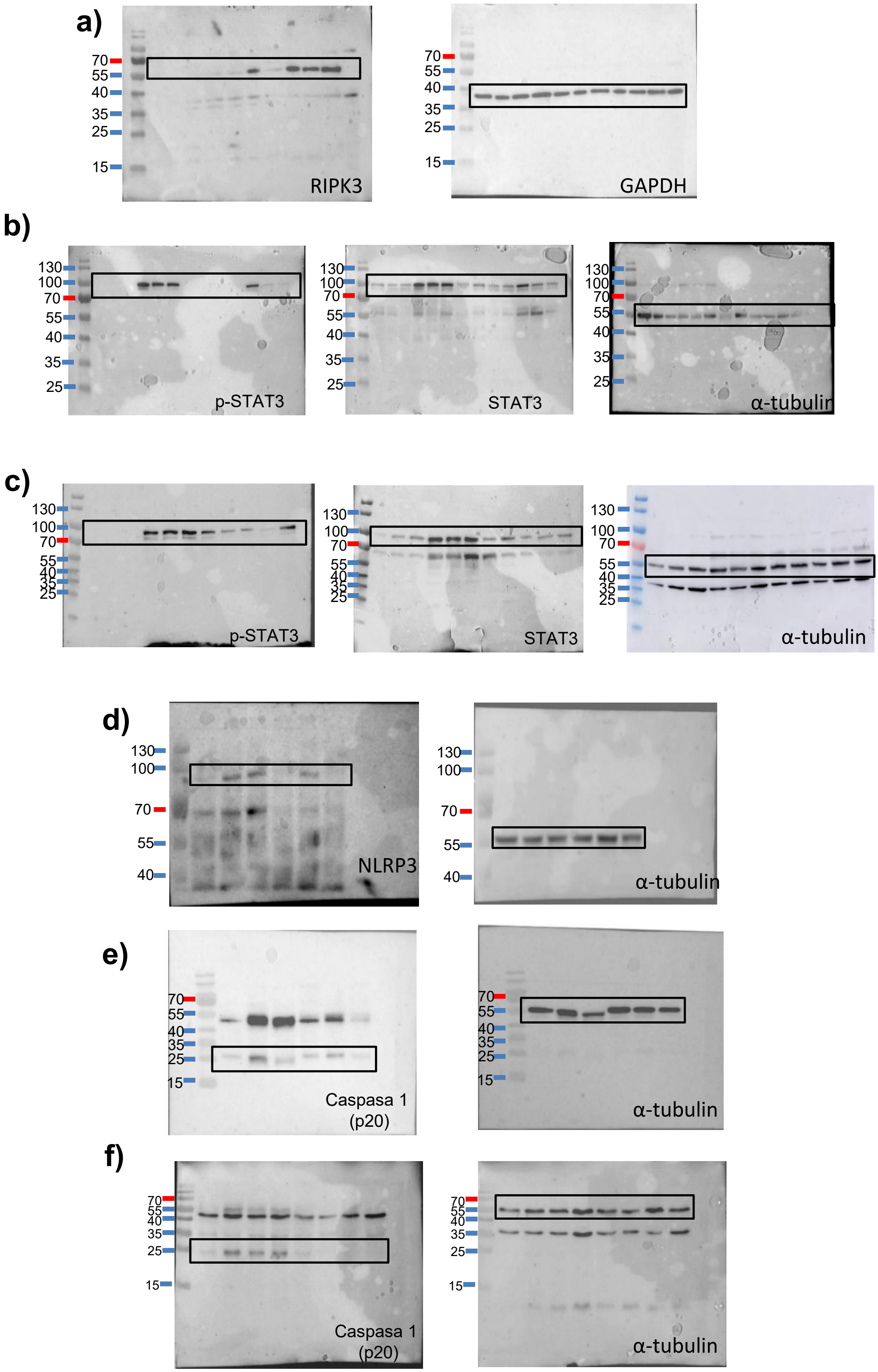

Uncropped gel scans merged with molecular weight images for all presented western blots. A) Corresponds to Fig. 3.B. B) Corresponds to Fig. 6.B. C) Corresponds to Fig. 6.G. D) Corresponds to SF. 3.A. E) Corresponds to SF. 3.B. F) Corresponds to SF. 3.C
