# Supplementary material for: The RIPK3-IL-6 axis mediates kidney injury in cytokine storm syndrome
Source: Cell Death Dis. 2026 Apr 15;17(1):714. doi: 10.1038/s41419-026-08686-1 (PMC13470091; doi:10.1038/s41419-026-08686-1)
Supplement: Supplementary file 3 [file 41419_2026_8686_MOESM3_ESM.pdf]

SPRINGER NATURE

Corresponding Author Name: Ana B. Sanz
Manuscript Number: CDDIS-25-569

Reporting Summary

Springer Nature wishes to improve the reproducibility of the work that we publish. This checklist is used to ensure good reporting standards and to improve the reproducibility. Please respond completely to all questions relevant to your manuscript. For more information, please read the journal's Guide to Authors.

☐ **Check here to confirm that the following information is available in the Material & Methods section:**

- **The exact sample size (*n*)** for each experimental group/condition, given as a number, not a range
- **A description of the sample collection** allowing the reader to understand whether the samples represent technical or biological replicates (including how many animals, litters, culture, etc.)
- **A statement of how many times the experiment shown was replicated in the laboratory**
- **Definitions of statistical methods and measures**: For small sample sizes (n<5) descriptive statistics are not appropriate, instead plot individual data points
  - Very common tests, such as *t*-test, simple χ^2 tests, Wilcoxon and Mann-Whitney tests, can be unambiguously identified by name only, but more complex techniques should be described in the methods section
  - Are tests one-sided or two-sided?
  - Are there adjustments for multiple comparisons?
  - **Statistical test results**, e.g., ***P* values**
  - Definition of **'center values'** as **median or mean**;
  - Definition of **error bars as s.d. or s.e.m. or c.i.**

Please ensure that the answers to the following questions are reported in the manuscript itself. We encourage you to include a specific subsection in the methods section for statistics, reagents and animal models. Below, provide the page number or section and paragraph number.

Statistics and general methods	Reported in section/paragraph or page #
1. How was the sample size chosen to ensure adequate power to detect a pre-specified effect size? (Give section/paragraph or page #)	For in vitro experiments, the sample size was based on effect sizes reported in previous studies. No formal sample size calculation was performed; the
For animal studies, include a statement about sample size estimate even if no statistical methods were used.	The sample size for the animal models was calculated using the application provided by the Institutional Animal Care and Use Committee (University of Illinois), which predicts
2. Describe inclusion/exclusion criteria if samples or animals were excluded from the analysis. Were the criteria pre-established? (Give section/paragraph or page #)	No data were excluded.
3. If a method of randomization was used to determine how samples/animals were allocated to experimental groups and processed, describe it. (Give section/paragraph or page #)	There was no randomization for in vitro assays. For in vivo assays see below.
For animal studies, include a statement about randomization even if no randomization was used.	For animal studies, siblings were distributed randomly in different conditions by the animal facility staff.

4. If the investigator was blinded to the group allocation during the experiment and/or when assessing the outcome, state the extent of blinding. (Give section/paragraph or page #)	There was no blinding for in vitro assays. For in vivo assays see below.
For animal studies, include a statement about blinding even if no blinding was done.	Histological and immunohistochemical assessments were performed in a blinded manner (method section, page 15
5. For every figure, are statistical tests justified as appropriate?	Yes; Means among three or more groups were compared using ANOVA with Tukey'spost-hoc test, one-way ANOVA
Do the data meet the assumptions of the tests (e.g., normal distribution)?	Normal distributions were tested using the Shapiro-Wilk test previously to the statistical analyses. All assays' data
Is there an estimate of variation within each group of data?	All histograms in the article show the standard deviations of each group of data as error bars.
Is the variance similar between the groups that are being statistically compared? (Give section/paragraph or page #)	Brown-Forsythe tests were performed to verify that differences among the variances of each condition were not significant, except for the data shown in Figures 1F, 1G,

Reagents

	Reported in section/paragraph or page #
6. Report the source of antibodies (vendor and catalog number)	Antibody information is provided in methods section (page 16-17).
7. Identify the source of cell lines and report if they were recently authenticated (e.g., by STR profiling) and tested for mycoplasma contamination	The source of the cell lines is stated in methos section (page 18). The cells are routinely tested for mycoplasma by PCR, but this is not indicated in the manuscript.

Animal Models

	Reported in section/paragraph or page #
8. Report species, strain, sex and age of animals	It is indicated in method section, page 13.
9. For experiments involving live vertebrates, include a statement of compliance with ethical regulations and identify the committee(s) approving the experiments.	The statement of compliance with the ethical regulations for in vivo assays is included in page 13.

10. We recommend consulting the ARRIVE guidelines (*PLoS Biol.* **8**(6), e1000412,2010) to ensure that other relevant aspects of animal studies are adequately reported.

Human subjects

Reported in section/paragraph or page #

11. Identify the committee(s) approving the study protocol.	Not applicable.
12. Include a statement confirming that informed consent was obtained from all subjects.	Not applicable.
13. For publication of patient photos, include a statement confirming that consent to publish was obtained.	Not applicable.
14. Report the clinical trial registration number (at ClinicalTrials.gov or equivalent).	Not applicable.

15. For phase II and III randomized controlled trials, please refer to the CONSORT statement and submit the CONSORT checklist with your submission.

16. For tumor marker prognostic studies, we recommend that you follow the REMARK reporting guidelines.

Data deposition

Reported in section/paragraph or page #

17. Provide accession codes for deposited data. Data deposition in a public repository is mandatory for:<br>a. Protein, DNA and RNA sequences<br>b. Macromolecular structures<br>c. Crystallographic data for small molecules<br>d. Microarray data	The data of RNA-seq in CSS-AKI have been deposited in NCBI GEO database with accession number GSE243633 (page 25).

Deposition is strongly recommended for many other datasets for which structured public repositories exist; more details on our data policy are available in the Guide to Authors. We encourage the provision of other source data in supplementary information or in unstructured repositories such as Figshare and Dryad. We encourage publication of Data Descriptors (see Scientific Data) to maximize data reuse.

18. If computer code was used to generate results that are central to the paper's conclusions, include a statement in the Methods section under "**Code availability**" to indicate whether and how the code can be accessed. Include version information as necessary and any restrictions on availability.	Only commercial software was employed in this study. The supplier was indicated when such software was mentioned (methods section).
